# Dementia Subtypes Defined Through Neuropsychiatric Symptom–Associated Brain Connectivity Patterns

**DOI:** 10.1001/jamanetworkopen.2024.20479

**Published:** 2024-07-08

**Authors:** Kanhao Zhao, Hua Xie, Gregory A. Fonzo, Nancy B. Carlisle, Ricardo S. Osorio, Yu Zhang

**Affiliations:** 1 Department of Bioengineering, Lehigh University, Bethlehem, Pennsylvania; 2 Center for Neuroscience Research, Children’s National Hospital, Washington, District of Columbia; 3 George Washington University School of Medicine, Washington, District of Columbia; 4 Center for Psychedelic Research and Therapy, Department of Psychiatry and Behavioral Sciences, Dell Medical School, University of Texas at Austin; 5 Department of Psychology, Lehigh University, Bethlehem, Pennsylvania; 6 Department of Psychiatry, New York University Grossman School of Medicine, New York, New York; 7 Department of Electrical and Computer Engineering, Lehigh University, Bethlehem, Pennsylvania

## Abstract

**Importance:**

Understanding the heterogeneity of neuropsychiatric symptoms (NPSs) and associated brain abnormalities is essential for effective management and treatment of dementia.

**Objective:**

To identify dementia subtypes with distinct functional connectivity associated with neuropsychiatric subsyndromes.

**Design, Setting, and Participants:**

Using data from the Open Access Series of Imaging Studies-3 (OASIS-3; recruitment began in 2005) and Alzheimer Disease Neuroimaging Initiative (ADNI; recruitment began in 2004) databases, this cross-sectional study analyzed resting-state functional magnetic resonance imaging (fMRI) scans, clinical assessments, and neuropsychological measures of participants aged 42 to 95 years. The fMRI data were processed from July 2022 to February 2024, with secondary analysis conducted from August 2022 to March 2024. Participants without medical conditions or medical contraindications for MRI were recruited.

**Main Outcomes and Measures:**

A multivariate sparse canonical correlation analysis was conducted to identify functional connectivity–informed NPS subsyndromes, including behavioral and anxiety subsyndromes. Subsequently, a clustering analysis was performed on obtained latent connectivity profiles to reveal neurophysiological subtypes, and differences in abnormal connectivity and phenotypic profiles between subtypes were examined.

**Results:**

Among 1098 participants in OASIS-3, 177 individuals who had fMRI and at least 1 NPS at baseline were included (78 female [44.1%]; median [IQR] age, 72 [67-78] years) as a discovery dataset. There were 2 neuropsychiatric subsyndromes identified: behavioral (*r* = 0.22; *P* = .002; *P* for permutation = .007) and anxiety (*r* = 0.19; *P* = .01; *P* for permutation = .006) subsyndromes from connectivity NPS–associated latent features. The behavioral subsyndrome was characterized by connections predominantly involving the default mode (within-network contribution by summed correlation coefficients = 54) and somatomotor (within-network contribution = 58) networks and NPSs involving nighttime behavior disturbance (*R* = −0.29; *P* < .001), agitation (*R* = −0.28; *P* = .001), and apathy (*R* = −0.23; *P* = .007). The anxiety subsyndrome mainly consisted of connections involving the visual network (within-network contribution = 53) and anxiety-related NPSs (*R* = 0.36; *P* < .001). By clustering individuals along these 2 subsyndrome-associated connectivity latent features, 3 subtypes were found (subtype 1: 45 participants; subtype 2: 43 participants; subtype 3: 66 participants). Patients with dementia of subtype 3 exhibited similar brain connectivity and cognitive behavior patterns to those of healthy individuals. However, patients with dementia of subtypes 1 and 2 had different dysfunctional connectivity profiles involving the frontoparietal control network (FPC) and somatomotor network (the difference by summed *z* values was 230 within the SMN and 173 between the SMN and FPC for subtype 1 and 473 between the SMN and visual network for subtype 2) compared with those of healthy individuals. These dysfunctional connectivity patterns were associated with differences in baseline dementia severity (eg, the median [IQR] of the total score of NPSs was 2 [2-7] for subtype 3 vs 6 [3-8] for subtype 1; *P* = .04 and 5.5 [3-11] for subtype 2; *P* = .03) and longitudinal progression of cognitive impairment and behavioral dysfunction (eg, the overall interaction association between time and subtypes to orientation was *F* *=* 4.88; *P* = .008; using the time × subtype 3 interaction item as the reference level: β = 0.05; *t* = 2.6 for time × subtype 2; *P* = .01). These findings were further validated using a replication dataset of 193 participants (127 female [65.8%]; median [IQR] age, 74 [69-77] years) consisting of 154 newly released participants from OASIS-3 and 39 participants from ADNI.

**Conclusions and Relevance:**

These findings may provide a novel framework to disentangle the neuropsychiatric and brain functional heterogeneity of dementia, offering a promising avenue to improve clinical management and facilitate the timely development of targeted interventions for patients with dementia.

## Key Points

**Question:**

Are specific patterns of brain dysfunction associated with more severe neuropsychiatric symptoms in some patients with dementia?

**Findings:**

In this cross-sectional study of 370 older adults, robust behavioral and anxiety neuropsychiatric subsyndromes were identified, with associations between brain functional connectivity and neuropsychiatric symptoms. Subsequent clustering revealed 3 dementia subtypes, with 1 resembling connectivity patterns of healthy individuals and others showing dysfunctional connectivity patterns associated with varying severity and progression of cognitive and behavioral impairment.

**Meaning:**

The capacity to combine brain and clinical metrics to distinguish variation in baseline dementia severity and progression of cognitive and behavioral impairment across subtypes may provide an empirical framework for enhancing clinical management and developing targeted interventions in dementia.

## Teaser

This cross-sectional study investigates the association of dementia subtypes defined by functional connectivity with neuropsychiatric symptom severity.

## Introduction

Dementia is highly heterogeneous, as reflected by variability in genetic risk factors, neuropsychiatric symptoms (NPSs), neuroimaging markers, comorbidities, and copathology.^1,2^ Conventional analyses of dementia using neuroimaging and psychological and behavior assessments have focused on characterizing case control group differences in comparison with healthy control groups, assuming homogeneity among patients with dementia.^3,4,5^ However, this approach neglects the inherent heterogeneity of NPSs and brain abnormalities of dementia, limiting our understanding of the underlying mechanisms of dementia.

To disentangle the heterogeneity of NPSs in dementia, previous studies analyzed neuropsychiatric subsyndromes clustered from NPSs.^6^ These subsyndromes provided insights into the pathophysiology of cognitive impairments in different dementia types^7^ and the effectiveness of some dementia treatments.^8,9,10^ However, the traditional subsyndrome analytic framework, using matrix decomposition of NPSs to obtain subsyndromes^7,11,12^ and comparing their differences across dementia categories,^4,13^ has 2 limitations. First, the matrix decomposition used in these analyses may not fully capture the complexity of NPSs or their neurobiological correlations. Second, typical dementia diagnostic categories derived from molecular phenotypes^14^ may not adequately reflect the heterogeneity of NPSs, aligning well with underlying neurobiology observed in patients with dementia.^4^

Recently, researchers have used data-driven multivariate methods to identify associations between neuroimaging features and psychological assessments for disentangling the shared heterogeneity across these 2 modalities.^15,16,17,18^ In 2 studies,^15,16^ sparse canonical correlation analysis (sCCA) or partial least squares analysis were used to uncover latent subsyndromes by associating phenotypes with functional connectivity (FC), which quantifies synchronization in resting-state functional resonance magnetic imaging (fMRI) signals among different brain regions. Using a similar method to derive subsyndromes for depression or autism, 2 additional studies^17,18^ further defined subtypes to parse the neurobiological heterogeneity present within these conditions. However, the lack of extended validation limits the generalizability of identified subsyndromes. More critically, no study to our knowledge has successfully derived FC-informed neuropsychiatric subsyndromes or identified potential subtypes by using the association between FC and NPSs, thus limiting our understanding of the heterogeneity within dementia.

In this study, we used sCCA^19^ to identify cross-validated, FC-informed NPS dimensions (subsyndromes) across individuals with dementia and those who were healthy. Furthermore, we investigated stable and replicable subtypes based on identified subsyndromes to elucidate dementia heterogeneity. Specifically, including 177 participants in the Open Access Series of Imaging Studies-3 (OASIS-3) as a discovery dataset, we associated FC and NPSs using sCCA. Then, k-means clustering was used to identify subtypes based on the obtained FC latent features from sCCA. To gain deeper insight into the psychological underpinnings of dementia of these subtypes, we compared NPSs, FC, and the baseline and longitudinal severity of cognitive-behavioral dysfunction among dementias of the 3 subtypes. Finally, main findings were validated on a replication dataset.

## Methods

**Figure 1.**
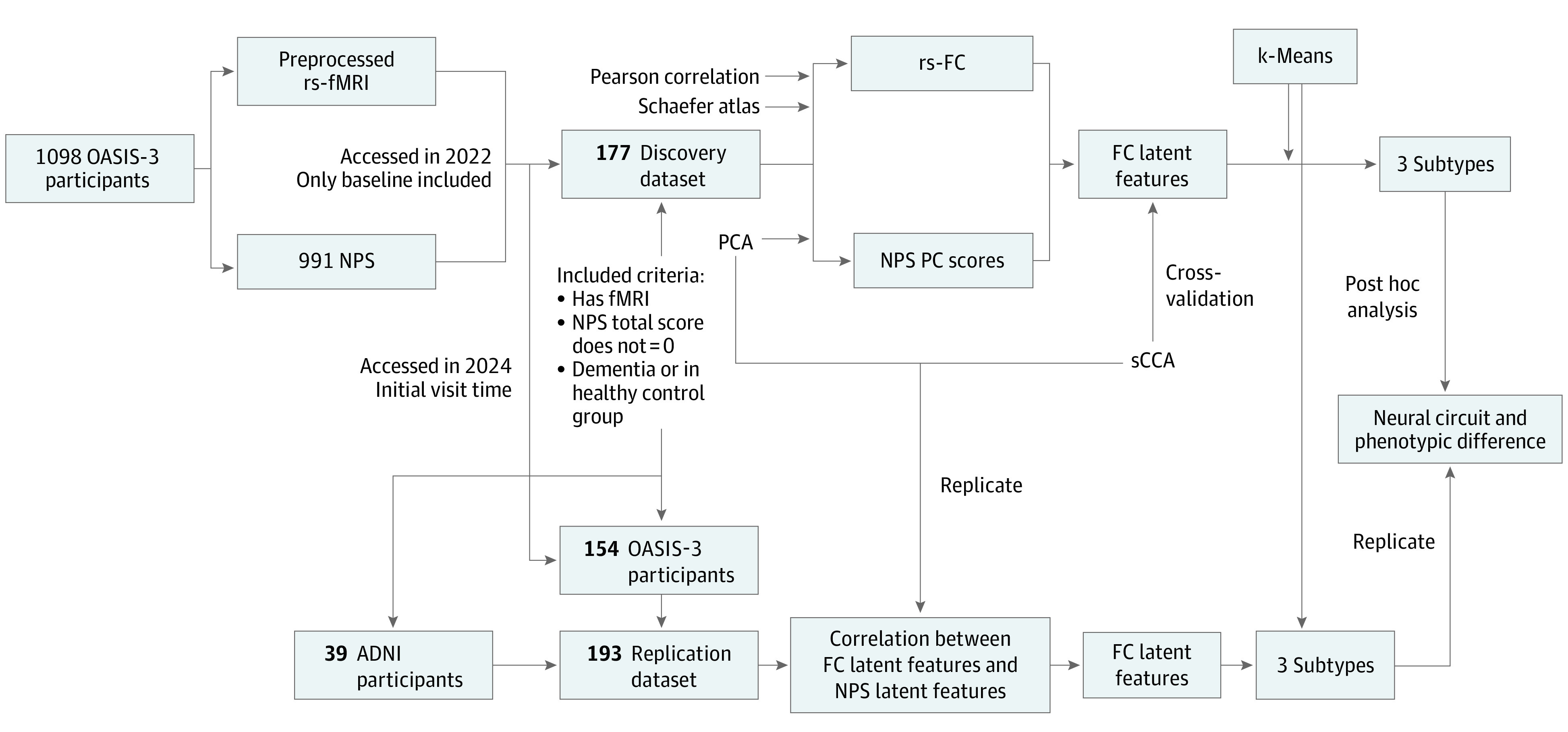
Study Flowchart The initial visit time means that the earliest enrolling date of the participant could be baseline or later sessions. Blood oxygen level–dependent signals extracted from the preprocessed functional magnetic resonance imaging (fMRI) were grouped into 100 regions of interests defined by Schaefer parcellation.^20^ Functional connectivity (FC) was obtained by calculating the Pearson correlation between pairwise region-of-interest time signals. ADNI indicates Alzheimer Disease Neuroimaging Initiative; NPS, neuropsychiatric symptom; OASIS-3, Open Access Series of Imaging Studies-3; PC, principal component; rs, resting state; sCCA, sparse canonical correlation analysis.

Participants from the OASIS-3 dataset provided informed consent in accordance with procedures approved by the institutional review board (IRB) of Washington University. The Alzheimer Disease Neuroimaging Initiative (ADNI) study was reviewed by the IRB at each participating site (see full list of IRBs in the eMethods in Supplement 1). This cross-sectional study was not reviewed by the IRB because it involved only a secondary analysis of these 2 public datasets. This study followed the Strengthening the Reporting of Observational Studies in Epidemiology (STROBE) reporting guideline. The proposed analytical framework for this study is illustrated in Figure 1.^20^

### Discovery Dataset

In this study, we used data from the OASIS-3 database, which enrolled participants at Washington University in St Louis over 15 years.^21^ A total of 1098 participants aged 42 to 95 years were enrolled beginning in 2005. Exclusion criteria included medical conditions or medical contraindications for the study groups. We performed the secondary analysis on the discovery dataset, including 177 participants with resting-state fMRI and at least 1 NPS at baseline from OASIS-3 from August 2022 to August 2023. Some scanning parameters for fMRI were a repetition time (TR) of 2.2 seconds, an echo time (TE) of 27 ms, and a flip angle (FA) of 90°. Acquired fMRI data were preprocessed using the fMRIPrep pipeline,^22^ followed by FC calculation using Pearson correlation (eMethods in Supplement 1).

### Replication Dataset

We reaccessed the OASIS-3 cohort in March 2024, incorporating 154 newly released participants with fMRI and NPS data into our replication dataset. The ADNI is an observational program, which enrolled participants aged 55 to 90 years to analyze biomarkers related to AD,^23^ that launched in 2003. The participant recruitment for ADNI was approved by the IRB of each participating site (see full list of IRBs in the eMethods in Supplement 1). Exclusion criteria comprised current use of psychoactive medication and a history of various mental disorders.^23^ We accessed the public dataset in 2024 and integrated 39 participants with fMRI and NPS data as part of our replication dataset. To match the diagnostic information in the OASIS-3 cohort, patients diagnosed with mild cognitive impairment were excluded. We performed the secondary analysis on the replication dataset in March 2024. Some scanning parameters for fMRI were a TR of 3.0 seconds, a TE of 32 ms, and an FA of 50°.

### Clinical and Psychological Assessments

The Neuropsychiatric Inventory^24^ is commonly used for evaluating NPSs. It consists of 12 domains: delusions, hallucinations, agitation, depression, anxiety, euphoria, apathy, disinhibition, irritability, aberrant motor behavior, nighttime behavior disturbance, and appetite abnormality. The Neuropsychiatric Inventory assesses symptoms in 4 levels: 0 (no symptoms), 1 (mild but not significant change), 2 (significant but not dramatic change), and 3 (dramatic change). Additionally, to assess functional impairment and cognitive-behavioral dysfunction, multiple measures were used: the Clinical Dementia Rating Scale (CDR), Mini-Mental State Examination (MMSE), Functional Activities Questionnaire (FAQ), and neuropsychological assessment battery^25^ (eMethods in Supplement 1).

### Statistical Analysis

#### Connectivity NPS–Associated Dimension Analysis

The significance level used in this study was *P* < .05 from 2-sided tests in general. Only *P* values from the permutation test were 1-sided. Significant correlations (*P* < .05 in a 2-sided test against the alternative hypothesis that *r* ≠ 0) among NPSs observed in a previous study^1^ and in our study (eFigure 1 in Supplement 1) raised concerns about ill conditioning for the subsequent canonical correlation analysis step due to collinearity.^26^ To address this issue, we used principal component analysis (PCA) to decompose collinear features into 7 orthogonal principal components from 12 NPS domains (eFigure 2 in Supplement 1), with more than 80% of variance explained.

**Figure 2.**
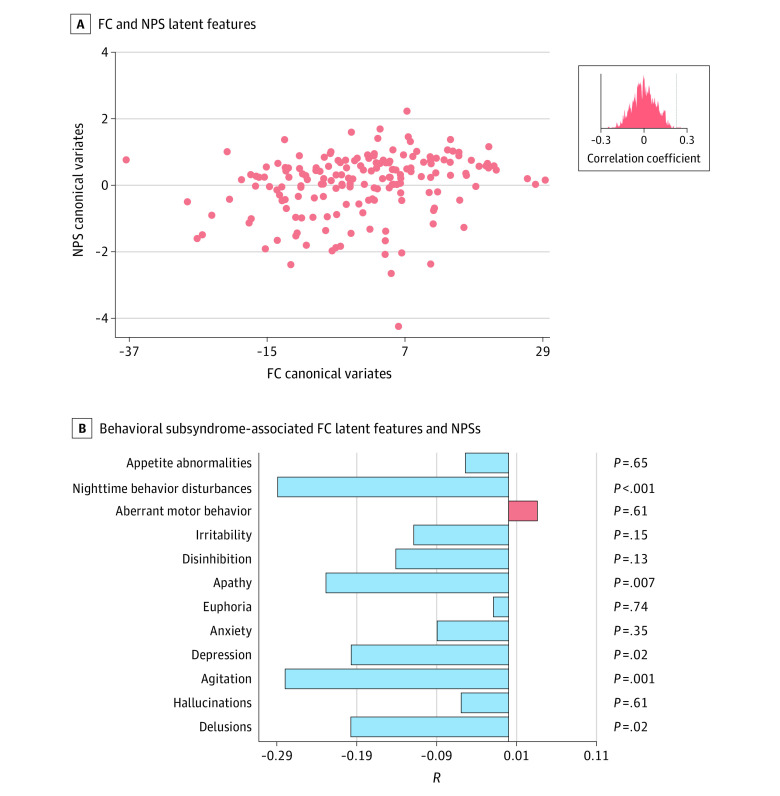
Behavioral Subsyndrome Profile and Associated Functional Connectivity (FC) Pattern A, Correlation between FC and neuropsychiatric symptom (NPS) latent features evaluated by 10-fold cross-validation transformed from sparse canonical correlation analysis is shown. Dots indicate latent feature values of each participant. The inset distribution plot shows Pearson correlation coefficients between NPS and FC canonical variates from 1000 permuted sparse canonical correlation analysis models. B, Correlation coefficients between behavioral subsyndrome–associated FC latent features and NPSs are shown.

**Figure 3.**
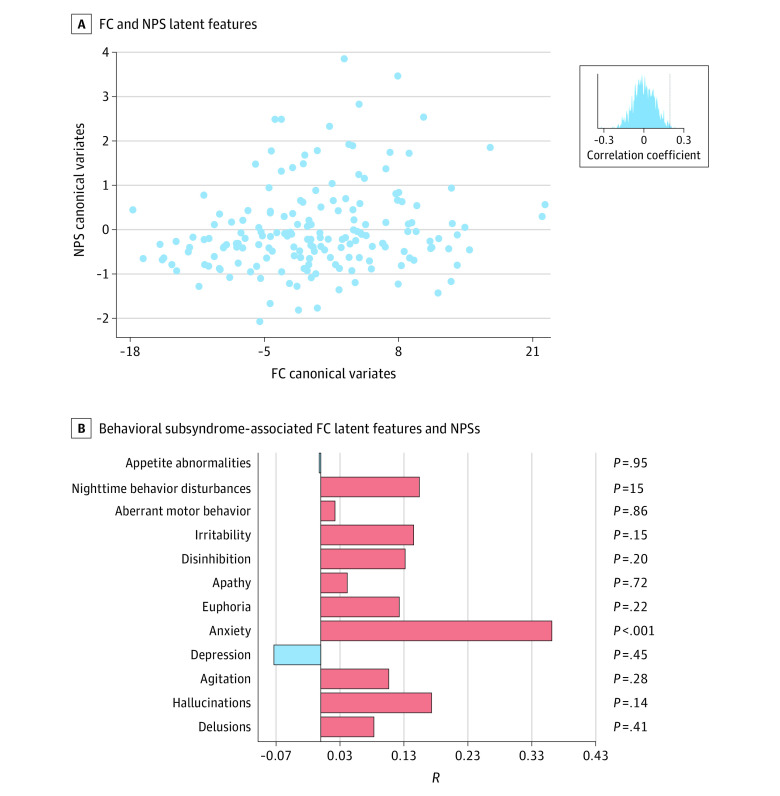
Anxiety Subsyndrome Profile and Associated Functional Connectivity (FC) Pattern A, Correlation between FC and neuropsychiatric symptom (NPS) latent features evaluated by 10-fold cross-validation is shown. B, Correlation coefficients between anxiety subsyndrome–associated FC latent features and NPSs are shown.

We used sCCA in the PMA package version 1.2.1 from R statistical software version 4.1.2 (R Project for Statistical Computing) to identify canonical variates with maximal correlation between the principal components of NPSs and FC across healthy individuals in the control group and patients, alleviating the overfitting issues through *L*_1_ regularization.^27^ Model performance was assessed by calculating the mean Pearson correlation of pairwise canonical variates in 10-fold cross-validation (1-sided test against the alternative hypothesis that *r* > 0). The highest mean correlation was achieved with 7 principal components and an *L*_1_ regularization penalty of 0.6 (eFigure 2 in Supplement 1). For subsequent analyses, we focused on the first and second canonical variates because they accounted for nearly 80% covariance between FC and NPSs (eFigure 2 in Supplement 1) and exhibited significant correlation in the test set, surviving a permutation test with false discovery rate correction (Figure 2A and Figure 3A; eTable 1 in Supplement 1).

#### Clustering-Based Identification of Neurophysiological Subtypes

Using neuropsychiatric subsyndrome–associated latent FC features, we used k-means clustering to identify distinct neurophysiological subtypes in dementia. Considering the shared phenotypes between patients with dementia and healthy individuals in the control group, our clustering analysis incorporated both groups.^28^ We assessed clustering performance by calculating Calinski-Harabasz and Silhouette metrics in 1000 trials of random subsamples of 90% of participants^29,30^ and tested cluster assignment stability by calculating the consistency of pairs of participants across 1000 random subsamplings. To ensure the robustness of clusters, we conducted hierarchical clustering (Ward method) and compared subtypes with those obtained using k-means clustering.

#### Post Hoc Analyses

We examined contributions of original FC and NPSs to the identified neuropsychiatric subsyndromes, calculating Pearson correlation coefficients between FC and subsyndrome-associated FC latent features and Spearman correlation coefficients between NPS scores and subsyndrome-associated NPS latent features. Additionally, we used Pearson correlation and analysis of variance to examine associations between phenotypic characteristics and FC latent features.

To investigate neural circuit abnormalities within each subtype, we compared FC between patients with dementia in each subtype and healthy individuals in the control group using the Wilcoxon signed-rank test (false discovery rate correction). The 2-tailed χ^2^ test, Kruskal-Wallis analysis, and Dunn test were used to detect differences in phenotypical variables across dementias of each subtype. To examine longitudinal cognitive progression within each subtype, linear mixed-effects models were used. Dependent variables included phenotypic measures at each study visit, and independent variables included subtype label, time state, and the interaction of subtype label.

Statistical analyses in this study were implemented using scipy version 1.11.1, statsmodels version 0.12.2, and sklearn version 1.1.3 packages in Python programming language version 3.9 (Python Software Foundation). Linear mixed-effects models and clustering were implemented in Matlab version 2022b (MathWorks). The main code for the analysis of this study has been released at GitHub.^31^ We processed fMRI data from July 2022 to February 2024 and performed secondary analysis from August 2022 to March 2024.

#### Replication Analysis

To verify whether our findings from the discovery cohort were generalizable, we used the obtained PCA and sCCA models to transform FC and NPSs into latent space and calculated the correlation between their latent features in the replication dataset. We then used subtype centroids of the discovery dataset as initialization points to implement k-means clustering on FC latent features of participants from the replication dataset. Finally, the same post hoc statistical methods used in the discovery dataset were used to compare differences in FC and neuropsychological measurements between dementia subtypes in the replication dataset.

## Results

### Baseline Characteristics

A total of 177 participants in the OASIS-3 cohort (78 female [44.1%]; median [IQR] age, 72 [67-78] years) constituted the discovery dataset, while 154 newly released participants from OASIS-3 and 39 participants from ADNI constituted our replication dataset of 193 individuals (127 female [65.8%]; median [IQR] age, 74 [69-77] years). Demographic information can be found in eTables 2 to 4 in Supplement 1. In the discovery dataset, 102 individuals had dementia (38 female [37.3%]; median [IQR] age, 74 [72-76] years, while in the replication dataset, 88 individuals had dementia (62 female [70.5%]; median [IQR] age, 77 [72-81] years).

### Behavioral Subsyndrome and Associated FC Pattern

The first latent component showed a correlation between FC and NPS latent features (*r* = 0.22; *P* = .002; *P* for permutation = .007) (Figure 2A), as confirmed through 10-fold cross-validation. Following the finding in a previous study,^32^ we named the first latent component *behavioral subsyndrome* based on its negative correlation with NPSs of nighttime behavior disturbance (*R* = −0.29; *P* < .001), agitation (*R* = −0.28; *P* = .001), and apathy (*R* = −0.23; *P* = .007) (Figure 2B). Correlation coefficients and canonical weights of FC related to behavioral subsyndrome–associated FC latent features were visualized (eFigures 3 and 4 in Supplement 1). These results consistently indicated that behavioral subsyndrome–associated FC mainly involved connectivity within the somatomotor network (SMN; within-network contribution by summed absolute correlation coefficients = 58) and default mode network (DMN; within-network contribution = 54), as well as between-network connectivity of the frontoparietal control network (FPC), SMN, dorsal attention network (DAN), and DMN. Additionally, FC latent features exhibited positive correlations with total scores of MMSE and the category fluency test (FLU) of vegetable names (eMethods and eTable 5 in Supplement 1) and a negative correlation with CDR sum of boxes (eTable 6 in Supplement 1). A higher behavioral subsyndrome score was associated with lower scores across various CDR domains and less difficulty in paying attention as evaluated by FAQ (eTable 6 and eFigures 5 and 6 in Supplement 1).

### Anxiety Subsyndrome and Associated FC Pattern

The second latent component also showed a correlation between FC and NPS latent features (*r* = 0.19; *P* = .01; *P* for permutation = .006) (Figure 3A). This latent component was designated as the *anxiety subsyndrome* given that it showed the highest positive correlation with the NPS anxiety score (*R* = 0.36; *P* < .001) (Figure 3B). The contribution of FC to the anxiety subsyndrome was measured by correlation coefficients and canonical weights of FC (eFigures 3 and 4 in Supplement 1), revealing that the anxiety subsyndrome was dominantly derived from FC within the visual network (VIS; within-network contribution = 53) and across the VIS, SMN and DMN. Furthermore, the anxiety subsyndrome score correlated positively with the CDR sum of boxes score and FAQ paying attention score and correlated negatively with the MMSE, FLU of vegetable names, FLU of animal names, and Digit Symbol Coding in Wechsler Memory Scale scores (eMethods, eTables 6 and 5, and eFigure 6 in Supplement 1). A larger anxiety subsyndrome score was associated with increased CDR scores across 5 domains (eTable 5 and eFigure 5 in Supplement 1). Males exhibited significantly larger anxiety subsyndrome scores compared with females (eTable 7 in Supplement 1).

### Behavioral and Anxiety Subsyndrome–Associated FC Latent Features and Defined Subtypes

**Figure 4.**
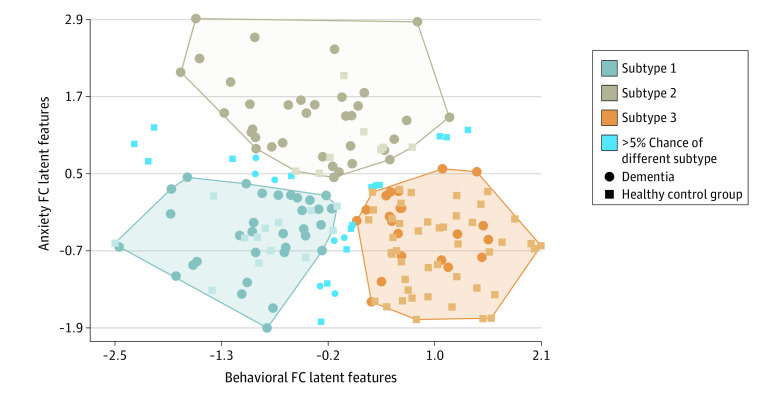
Subtypes Defined From Neuropsychiatric Symptom Subsyndrome–Associated Functional Connectivity (FC) Latent Features The scatter plot shows 3 clusters of participants along the dimension of the anxiety subsyndrome and behavioral subsyndrome. The x- and y-axis give sparse canonical correlation analysis–transformed component scores for the FC domain of each participant. Blue shapes indicate participants who had a greater than 5% chance of being assigned to different subtype labels in 1000 random 90% subsampling trials.

Using anxiety and behavioral subsyndrome–associated FC latent features, we identified 3 distinct neurophysiological subtypes through k-means clustering (subtype 1: 45 participants; subtype 2: 43 participants; subtype 3: 66 participants) (Figure 4), with high separability and stability (eFigure 7 in Supplement 1). A total of 23 participants had a greater than 5% chance of being assigned to different clusters in 1000 trials of random subsampling. Furthermore, consistent subtypes identified from hierarchical clustering (eFigure 8 in Supplement 1) demonstrated the robustness of subtype identification.

Using the Wilcoxon rank sum test, we identified significantly abnormal FC in patients with dementia compared with healthy individuals in the control group. In subtype 1, the most abnormal FC compared with the control group was observed between the left insula and superior parietal cortex (eFigure 9 in Supplement 1). Differences in brain connections mainly involved within-network connections of the SMN (summed absolute *z* values = 230) and VAN (summed z values = 148) and between-network connections of the SMN and FPC (summed z values = 173) and SMN and DAN (summed z values = 187) (eFigure 9 in Supplement 1). In subtype 2, the most abnormal FC compared with the control group was observed between the left inferior temporal cortex and parietal cortex (eFigure 9 in Supplement 1). These differences mainly involved within-network connections of the VIS (summed z values = 391) and between-network connections of the SMN and VIS (summed z values = 473) and SMN and DMN (summed z values = 248) (eFigure 9 in Supplement 1). For subtype 3, no significant FC differences were observed compared with all healthy individuals in the control group.

We further observed significant differences in the severity of NPSs among patients with dementia across the 3 subtypes (eTable 8 and eFigure 10 in Supplement 1). Patients in subtypes 1 and 2 exhibited more severe total NPSs, subsyndromes, and functional impairment compared with patients in subtype 3. The median was compared using Dunn test; for example, the median [IQR] of the total score of NPSs was 2 [2-7] for subtype 3 vs 6 [3-8] for subtype 1 (*P* = .04) and 5.5 [3-11] for subtype 2 (*P* = .03). Additionally, the presence of greater levels of hallucination, anxiety, and apathy symptoms, along with the 2 distinct neuropsychiatric subsyndromes, distinguished dementia between subtypes 1 and 2. Linear mixed-effect models analyses indicated that dementia in subtype 2 showed significantly increased cognitive impairment over time in orientation (the overall interaction association of time by subtypes to orientation was *F* = 4.88; *P* = .008; using time × subtype 3 as the reference level, β = 0.05 and *t* = 2.6 for time × subtype 2; *P* = .01) and hobbies maintenance compared with subtype 3. (eFigure 11 in Supplement 1). Furthermore, the longitudinal progression of cognitive functional change in animal naming, digit writing, and dot connecting tests measured by the neuropsychological assessment battery revealed similar rates of decline in subtypes 1 and 2, which were worse than those in subtype 3 (eFigure 11 in Supplement 1). To further verify the uniqueness of subtypes defined from NPS-associated FC latent features, we clustered solely on NPS scores. We identified 2 clusters (eFigure 12 in Supplement 1). Abnormal FC and phenotypic patterns were different from clusters derived using NPS-associated FC latent features (eFigures 9 and 12 and eTables 8 and 9 in Supplement 1). Baseline FC latent features were correlated with longitudinal counterparts (eMethods and eFigure 13 in Supplement 1). In k-means clustering among patients with dementia, subtype 1 was relatively stable but subtype 3 had an increased number of patients with dementia (eMethods and eFigure 14 in Supplement 1). In principal component analysis, we found that 6 or 8 principal components were associated with a decrease in mean correlations between FC and NPS latent features compared with 7 components (eMethods and eFigure 15 in Supplement 1).

### Replication Analysis

We validated our findings using a replication dataset, using established PCA and sCCA models (eFigure 16 in Supplement 1). Correlations were observed between FC latent features and NPS latent features for behavioral and anxiety subsyndromes. Compared with all healthy individuals in the control group, patients with dementia of subtype 1 exhibited abnormal connections in the SMN, VIS, and FPC, while those with subtype 2 showed abnormalities in the SMN, VIS, and DMN. No significantly abnormal FC was observed in patients with dementia of subtype 3. Additionally, patients with dementia of subtype 2 exhibited the most severe impairments across various CDR and FAQ domains compared with patients in subtype 3 (eFigure 16 and eTable 10 in Supplement 1). Findings from replication dataset were consistent with results identified from the discovery dataset.

## Discussion

Conducting sparse canonical correlation analysis in this cross-sectional study, we identified 2 robust subsyndromes, behavioral and anxiety, in the NPS-FC–correlated latent space, revealing distinct connectivity patterns and characteristic phenotypes, such as daily living disability. We then clustered participants into 3 subtypes based on NPS subsyndrome–associated FC latent features and found significant differences in clinical assessments, baseline connectivity patterns, and longitudinal clinical progression among dementia subtypes. Notably, patients with subtypes 1 and 2 exhibited more severe cognitive dysfunctions at baseline and greater deterioration in cognitive abilities over time compared with healthy individuals in the control group, while patients with subtype 3 displayed similar brain and cognitive phenotypes to healthy individuals in the control group. These findings underscore the potential for precise interventions tailored to the unique cognitive impairment and dysfunction observed in dementia of different subtypes.

### NPS Subsyndromes and Associated FC Patterns

In our study, the behavioral subsyndrome positively associated with better cognitive performance, while the anxiety subsyndrome positively associated with cognitive impairment. This reflects the partial overlap between these subsyndromes in capturing neurobehavioral dimensions inversely given that the behavioral subsyndrome negatively correlated with delusion and depression and the anxiety subsyndrome positively correlated with hallucination and anxiety. Previous research reported positive correlations between depression and anxiety,^33^ as well as between delusions and hallucinations.^34^ Moreover, both subsyndromes were related to between-network connections involving VIS-SMN, DMN-SMN, and DMN-VIS connections, aligning with studies associating hallucinations and delusions with atrophy and decreased glucose metabolism of the visual network in patients with Alzheimer disease.^35^ Patients with Alzheimer disease or Parkinson disease have high metabolic activity in brain regions involving the DMN and SMN, which positively correlates with anxiety and depression symptoms.^36^ Our study’s results, along with these previously reported findings, support the notion that cognitive impairment, accompanied by delusion, hallucination, anxiety, and depression symptoms, is intricately associated with DMN circuitry and atrophy of the SMN and DMN.^37^

Our findings provide valuable insights into the distinct cognitive impairments associated with each neuropsychiatric subsyndrome. The behavioral subsyndrome specifically captured variance in instrumental activities of daily living involving business affairs and driving function deficits, which are sensitive in detecting mild dementia.^38,39^ Additionally, this subsyndrome correlated with connectivity within the SMN, known for its role in decision-making and driving ability.^40,41^ These findings suggest that the behavioral subsyndrome may be particularly sensitive in detecting mild cognitive impairment by capturing decision-making deficits associated with SMN dysfunctions. Furthermore, our study indicated a negative correlation between the anxiety subsyndrome and verbal fluency and delayed logical memory scores. This subsyndrome was primarily characterized by connections between the DMN and VAN and connections within the VIS. Previous studies have associated verbal fluency scores with white matter hyperintensities in the DMN of patients with dementia and activation of the VIS in healthy individuals in control groups.^42,43^ Moreover, decline in episodic memory performance has been associated with altered DMN connectivity in patients with cognitive impairment.^44^ In line with these findings, the anxiety subsyndrome may be the psychopathological dimension that more specifically captured dysfunction in episodic memory and language processing.

### Dementia Subtypes

Prior studies in identifying dementia subtypes have primarily focused on a single modality, including psychological measurements or structural imaging.^45,46^ To our knowledge, there has been a lack of studies that use latent features associated with NPSs and FC, which have emerged as biomarkers for early dementia detection.^47,48,49^ Our study revealed novel subtypes based on NPS-associated FC latent features, offering the potential to disentangle the heterogeneity of early dementia.

We observed shared connectivity differences in patients with dementia of subtypes 1 and 2 compared with healthy individuals in the control group, particularly within the VIS, between the FPC and SMN, and between the DMN and VAN. These network abnormalities were associated with deficits in social conduct, emotional processing, and episodic memory retrieval. Consequently, patients with dementia of subtypes 1 and 2 performed more poorly in orientation and hobby maintenance than patients with subtype 3 and healthy individuals in the control group. Additionally, dysfunctions in brain networks, such as the DMN, FPC, SMN, and VAN, have been associated with cognitive and behavioral abnormalities across various dementia types, including Alzheimer disease, frontotemporal dementia, and dementia with Lewy bodies.^14,50^ These findings suggest that these shared FC dysfunctions and cognitive abnormalities observed across our 2 dementia subtypes, alongside traditional dementia subtypes, may reflect a common cognitive impairment–related neuropathological mechanism.

At baseline, dementia in subtype 2 had more severe hallucination and anxiety symptoms than subtype 1, possibly associated with distinct connectivity patterns within the VIS and between the VIS and SMN given that hallucinations have been associated with pathological changes in the visual network.^51^ Visual dysfunction may contribute to emotional control–related neuropsychiatric symptoms, such as anxiety and apathy.^51,52^ Enhanced differences of FC involving the DMN in subtype 2, known for its role in emotion regulation, may explain this contribution. Moreover, as age increased, dementia in subtype 2 showed more severe cognitive and behavioral dysfunction than dementia in subtype 1. Previous research has suggested that visual impairment is associated with increased risk of cognitive impairment and incident dementia.^53^ Taken together, our findings suggest a plausible association between increased hallucinations and development of dementia-related cognitive impairment and emotional dysfunction.

### Limitations

There are some limitations and potential extensions to be considered. First, exploring additional biological factors, like genomics and proteome sequence, could enhance our understanding of dementia subtypes and their underlying mechanisms. Second, the generalizability of our identified subsyndromes and subtypes could be further validated in large, demographic-matched datasets to ensure their representation of the broader dementia population in clinical settings. Third, incorporating longitudinal imaging and clinical data in future studies may deepen insights into dementia’s progressive nature and the interplay between brain-psychopathology dimensions over time.

## Conclusions

This cross-sectional study offers important insights into dementia heterogeneity using a data-driven analytical framework. We identified behavioral and anxiety subsyndromes capturing distinctions shared between brain circuits and NPSs in dementia. The behavioral subsyndrome–associated FC latent features were specifically and negatively associated with dysfunction in daily activities related to taxes and business affairs, while anxiety subsyndrome–associated FC latent features were specifically and positively associated with memory and language deficits. Using these subsyndromes, our clustering analyses revealed 3 stable subtypes. Patients with dementia of 2 subtypes exhibited significant FC differences involving the DMN, FPC, and SMN associated with memory and emotional dysfunction at baseline and longitudinally. Patients with the third subtype resembled healthy individuals in the control group in cognitive function and FC. Importantly, these findings were replicated using an external replication dataset. Overall, our study provides a unique perspective on the complex heterogeneity of dementia and underlying neurobiological mechanisms, which may ultimately inform the development of targeted interventions and diagnostic tools for patients in different subtypes.

## Notes

Supplement 1.
eMethods.
**eFigure 1.** Correlation between pairs of neuropsychiatric symptoms **eFigure 2.** Hyperparameter selection **eFigure 3.** Top 50 (absolute) strongest correlations (FDR correction) between sCCA transformed FC latent features and FC features **eFigure 4.** Contribution of FCs to the first and second sCCA-transformed FC variates **eFigure 5.** Tukey honest significance test results for behavioral and anxiety subsyndromes **eFigure 6.** Tukey honest significance test of first and second canonical variates **eFigure 7.** Cluster evaluation analyses of k-means clustering **eFigure 8.** Cluster evaluation analyses of hierarchical clustering **eFigure 9.** Comparing FC of dementia in subtypes 1 and 2 with all healthy participants in control group **eFigure 10.** Dunn multiple comparison results of all ordinal clinical measurements **eFigure 11.** Differences in longitudinal change of various clinical scores among dementia in subtypes **eFigure 12.** Clustering on NPS scores only **eFigure 13.** Consistency in longitudinal changes of NPS subsyndrome–associated FC latent features **eFigure 14.** Association between subtypes clustering on participants including and excluding healthy control group **eFigure 15.** Principal components contribution to identification of subtypes **eFigure 16.** Replicability verified in the replication dataset **eTable 1.** Summary of correlation between third to seventh sCCA-transformed connectivity latent scores and NPS latent scores **eTable 2.** Characteristic information of participants from OASIS-3 and ADNI cohorts **eTable 3.** Characteristic information of participants from the discovery dataset **eTable 4.** Characteristic information of participants from the replication dataset **eTable 5.** Correlation between FC latent scores and continuous clinical measurements measured by using Pearson correlation **eTable 6.** Association between FC latent scores and scores of various categorical clinical measurements and biomarkers **eTable 7.** Associations between FC latent scores and demographic information **eTable 8.** Differences of various clinical measurements and demographic information across dementia in 3 subtypes defined from FC-associated latent features **eTable 9.** Difference of various clinical measurements and demographic information across dementia in subtypes defined from NPS **eTable 10.** Differences of various clinical measurements and demographic information across dementia in 3 subtypes defined from FC–associated latent features in the replication dataset
eReferences.

Supplement 2.
Data Sharing Statement
