# Supplementary material for: Dementia Subtypes Defined Through Neuropsychiatric Symptom–Associated Brain Connectivity Patterns
Source: JAMA Netw Open. 2024 Jul 8;7(7):e2420479. doi: 10.1001/jamanetworkopen.2024.20479 (PMC11231801; doi:10.1001/jamanetworkopen.2024.20479)
Supplement: Supplement 1. — eMethods. eFigure 1. Correlation between pairs of neuropsychiatric symptoms eFigure 2. Hyperparameter selection eFigure 3. Top 50 (absolute) strongest correlations (FDR correction) between sCCA transformed FC latent features and FC features eFigure 4. Contribution of FCs to the first and second sCCA-transformed FC variates eFigure 5. Tukey honest significance test results for behavioral and anxiety subsyndromes eFigure 6. Tukey honest significance test of first and second canonical variates eFigure 7. Cluster evaluation analyses of k-means clustering eFigure 8. Cluster evaluation analyses of hierarchical clustering eFigure 9. Comparing FC of dementia in subtypes 1 and 2 with all healthy participants in control group eFigure 10. Dunn multiple comparison results of all ordinal clinical measurements eFigure 11. Differences in longitudinal change of various clinical scores among dementia in subtypes eFigure 12. Clustering on NPS scores only eFigure 13. Consistency in longitudinal changes of NPS subsyndrome–associated FC latent features eFigure 14. Association between subtypes clustering on participants including and excluding healthy control group eFigure 15. Principal components contribution to identification of subtypes eFigure 16. Replicability verified in the replication dataset eTable 1. Summary of correlation between third to seventh sCCA-transformed connectivity latent scores and NPS latent scores eTable 2. Characteristic information of participants from OASIS-3 and ADNI cohorts eTable 3. Characteristic information of participants from the discovery dataset eTable 4. Characteristic information of participants from the replication dataset eTable 5. Correlation between FC latent scores and continuous clinical measurements measured by using Pearson correlation eTable 6. Association between FC latent scores and scores of various categorical clinical measurements and biomarkers eTable 7. Associations between FC latent scores and demographic information eTable 8. Diffe [file jamanetwopen-e2420479-s001.pdf]

Supplemental Online Content

Zhao K, Xie H, Fonzo GA, Carlisle NB, Osorio RS, Zhang Y. Dementia subtypes defined through neuropsychiatric symptom-associated brain connectivity patterns. *JAMA Netw Open*. 2024;7(7):e2420479. doi:10.1001/jamanetworkopen.2024.20479

eMethods.
eFigure 1. Correlation between pairs of neuropsychiatric symptoms
eFigure 2. Hyperparameter selection
eFigure 3. Top 50 (absolute) strongest correlations (FDR correction) between sCCA transformed FC latent features and FC features
eFigure 4. Contribution of FCs to the first and second sCCA-transformed FC variates
eFigure 5. Tukey honest significance test results for behavioral and anxiety subsyndromes
eFigure 6. Tukey honest significance test of first and second canonical variates
eFigure 7. Cluster evaluation analyses of k-means clustering
eFigure 8. Cluster evaluation analyses of hierarchical clustering
eFigure 9. Comparing FC of dementia in subtypes 1 and 2 with all healthy participants in control group
eFigure 10. Dunn multiple comparison results of all ordinal clinical measurements
eFigure 11. Differences in longitudinal change of various clinical scores among dementia in subtypes
eFigure 12. Clustering on NPS scores only
eFigure 13. Consistency in longitudinal changes of NPS subsyndrome–associated FC latent features
eFigure 14. Association between subtypes clustering on participants including and excluding healthy control group
eFigure 15. Principal components contribution to identification of subtypes
eFigure 16. Replicability verified in the replication dataset
eTable 1. Summary of correlation between third to seventh sCCA-transformed connectivity latent scores and NPS latent scores
eTable 2. Characteristic information of participants from OASIS-3 and ADNI cohorts
eTable 3. Characteristic information of participants from the discovery dataset
eTable 4. Characteristic information of participants from the replication dataset
eTable 5. Correlation between FC latent scores and continuous clinical measurements measured by using Pearson correlation
eTable 6. Association between FC latent scores and scores of various categorical clinical measurements and biomarkers
eTable 7. Associations between FC latent scores and demographic information
eTable 8. Differences of various clinical measurements and demographic information across dementia in 3 subtypes defined from FC-associated latent featurese
Table 9. Difference of various clinical measurements and demographic information across dementia in subtypes defined from NPS
eTable 10. Differences of various clinical measurements and demographic information across dementia in 3 subtypes defined from FC–associated

© 2024 Zhao K et al. *JAMA Network Open*.

latent features in the replication dataset
eReferences.

This supplemental material has been provided by the authors to give readers additional information about their work.

© 2024 Zhao K et al. *JAMA Network Open*.

Supplementary Materials

Supplementary methods

Institutional review boards of ADNI study

The Ethics committees/institutional review boards that approved the ADNI study are: Albany Medical Center Committee on Research Involving Human Subjects Institutional Review Board, Boston University Medical Campus and Boston Medical Center Institutional Review Board, Butler Hospital Institutional Review Board, Cleveland Clinic Institutional Review Board, Columbia University Medical Center Institutional Review Board, Duke University Health System Institutional Review Board, Emory Institutional Review Board, Georgetown University Institutional Review Board, Health Sciences Institutional Review Board, Houston Methodist Institutional Review Board, Howard University Office of Regulatory Research Compliance, Icahn School of Medicine at Mount Sinai Program for the Protection of Human Subjects, Indiana University Institutional Review Board, Institutional Review Board of Baylor College of Medicine, Jewish General Hospital Research Ethics Board, Johns Hopkins Medicine Institutional Review Board, Lifespan - Rhode Island Hospital Institutional Review Board, Mayo Clinic Institutional Review Board, Mount Sinai Medical Center Institutional Review Board, Nathan Kline Institute for Psychiatric Research & Rockland Psychiatric Center Institutional Review Board, New York University Langone Medical Center School of Medicine Institutional Review Board, Northwestern University Institutional Review Board, Oregon Health and Science University Institutional Review Board, Partners Human Research Committee Research Ethics, Board Sunnybrook Health Sciences Centre, Roper St. Francis Healthcare Institutional Review Board, Rush University Medical Center Institutional Review Board, St. Joseph's Phoenix Institutional Review Board, Stanford Institutional Review Board, The Ohio State University Institutional Review Board, University Hospitals Cleveland Medical Center Institutional Review Board, University of Alabama Office of the IRB, University of British Columbia Research Ethics Board, University of California Davis Institutional Review Board Administration, University of California Los Angeles Office of the Human Research Protection Program, University of California San Diego Human Research Protections Program, University of California San Francisco Human Research Protection Program, University of Iowa Institutional Review Board, University of Kansas Medical Center Human Subjects Committee, University of Kentucky Medical Institutional Review Board, University of Michigan Medical School Institutional Review Board, University of Pennsylvania Institutional Review Board, University of Pittsburgh Institutional Review

© 2024 Zhao K et al. *JAMA Network Open*.

Board, University of Rochester Research Subjects Review Board, University of South Florida Institutional Review Board, University of Southern, California Institutional Review Board, UT Southwestern Institution Review Board, VA Long Beach Healthcare System Institutional Review Board, Vanderbilt University Medical Center Institutional Review Board, Wake Forest School of Medicine Institutional Review Board, Washington University School of Medicine Institutional Review Board, Western Institutional Review Board, Western University Health Sciences Research Ethics Board, and Yale University Institutional Review Board.

© 2024 Zhao K et al. *JAMA Network Open*.

Clinical and psychological assessments

To assess functional impairment, we utilized multiple measures *Clinical Dementia Rating Scale (CDR), Mini-Mental State Examination (MMSE), and Functional Activities Questionnaire (FAQ).* The CDR evaluates cognitive impairment in six domains including memory, orientation, judgment and problem solving, community affair, home and hobbies and personal care, and provides a global CDR score and the sum of boxes (SOB) [1]. The CDR ranges from 0–3: no dementia (CDR = 0), questionable dementia (CDR = 0.5), MCI (CDR = 1), moderate cognitive impairment (CDR = 2), and severe cognitive impairment (CDR = 3). The MMSE is also designed for the evaluation of cognitive impairment [2], but only the total score was accessible. Smaller MMSE denotes more severe cognitive dysfunction. The functional ability is evaluated by FAQ [3] in 10 daily activities: 1) writing checks, paying bills, and keeping financial records; 2) assembling tax records and making out business and insurance papers; 3) shopping alone for clothes, household necessities, and groceries; 4) playing a game of skill such as bridge, other card game, or chess; 5) heating water for coffee or tea and turning off the stove; 6) preparing a balanced meal; 7) keeping track of current events; 8) paying attention to and understanding a TV program, book, or magazine; 9) remembering appointments, family occasions, and medications; and 10) travel out of the neighborhood. Moreover, the *neuropsychological assessment battery (NAB)* [4] was applied in assessing neuropsychiatric disturbances and cognitive and behavioral dysfunction. For the NAB (only accessed in OASIS-3 cohort), ten neuropsychological tests measure attention/working and episodic memory, executive function, and language, including the Digit Span Forward and Backward test, Logical Memory, and Digit Symbol Coding in Wechsler Memory Scale (WAIS-R) [5], Category fluency of animal and vegetable (FLU-ANI/ FLU-VEG) [6], Trail Making Test Part A and B [7], and Boston Naming Test [8]. The Digit Symbol Coding task from the Wechsler Adult Intelligence Scale is scored based on the number of correct symbol entries the participant makes within a set time limit. FLU was calculated the count of naming animal, vegetable item of corresponding category in one minute. No theoretical range reported from the ADNI and OASIS3 protocol.

MRI acquisition and preprocessing

Neuroimaging data in OASIS-3 was scanned in three different Siemens scanners (Siemens Vision 1.5 T, 2 scanners of TIM Trio 3T) and Siemens BioGraph mMR PET- MR 3T. High resolution T1-weighted structural image (TR = 2.4 s, TE = 3.08 ms, FOV = 256 × 256 mm, FA = 8°, voxel size 1 × 1 ×1 mm^3) and resting-state functional image (EPI; TR = 2.2 s, TE = 27 ms, FOV = 240 × 240 mm, FA = 90°, duration = 6 min, voxel size 4 × 4 × 4 mm, 36 slices) were used. Neuroimaging data in ADNI was scanned in 3T Philips system using magnetization-prepared rapid acquisition gradient echo[9]. Some scanner parameters of resting-state fMRI were: TR = 3.0 s, TE = 32 ms, FA = 50°, slice thickness = 2.5 mm.

The acquired rs-fMRI data were preprocessed using the reproducible fMRIPrep pipeline [10]. The T1 weighted image was corrected for intensity nonuniformity and then skull stripped. Spatial normalization was done through nonlinear registration, with the T1w reference [11]. Using FSL, brain tissue such as cerebrospinal fluid, white matter, and grey matter was segmented from the reference, brain-extracted T1 weighted image [12]. The fieldmap information was used to correct distortion in low-frequency and high-frequency components of fieldmap caused by field inhomogeneity. With less fieldmap distortion, a corrected echo-planar imaging reference was obtained from a more accurate co-registration with the anatomical reference. The blood oxygenation level dependent (BOLD) reference was then transformed to the T1-weighted image with a boundary-based registration method, configured with nine degrees of freedom to account for distortion remaining in the BOLD reference [13]. Head-motion parameters (rotation and translation parameters of volume-to-reference transform matrices) were estimated with MCFLIRT (FSL). BOLD signals were slice-time corrected and resampled onto the participant's original space with head-motion parameters, susceptibility distortion correction, and then resampled into standard space (MNI152NLin2009cAsym space), generating a preprocessed BOLD signal. Automatic removal of motion artifacts using independent component analysis (ICA-AROMA) [14] was performed on the preprocessed BOLD time-series in MNI space after removal of non-steady-state

© 2024 Zhao K et al. *JAMA Network Open*.

volumes and spatial smoothing with an isotropic Gaussian kernel of 6 mm FWHM (full-width half-maximum).

Sparse canonical correlation analysis (sCCA)

Canonical correlation analysis (CCA) is a multivariate statistical method used to explore the relationships between two sets of variables by identifying linear combinations that are maximally correlated across the two sets. It aims to find pairs of canonical variates that have maximum correlation with each other. Mathematically, given two sets of variables **X** and **Y**, CCA seeks to find weight vectors **a** and **b** such that the correlation between $\mathbf{Xa}$ and $\mathbf{Yb}$ is maximized. When confronted with high- dimensional data, traditional CCA may encounter overfitting issues due to the presence of irrelevant or redundant variables. Sparse CCA (sCCA) addresses this challenge by imposing sparsity constraints on the canonical weight vectors[15]. This is achieved by incorporating L_1 regularization into the optimization objective. This optimization problem can be expressed as: maximize $\mathbf{a}^{\mathrm{T}}\mathbf{X}^{\mathrm{T}}\mathbf{Yb}$ subject to $||\mathbf{a}||_2^2 \leq 1, ||\mathbf{b}||_2^2 \leq 1, ||\mathbf{a}||_1 \leq c_1, ||\mathbf{b}||_1 \leq c_2$. The optimization problem is solve using iterative algorithms[15], with the penalty parameters c_1 and c_2 controlling the L_1 norm. These parameters are fine-tuned through cross-validation grid search. In our specific application, where NPS scores serve as **Y** with low-dimensional features, we set c_2 to 1 to eliminate sparsity constraints, focusing solely on optimizing c_1 through cross-validation.

Model performance was assessed by calculating the average Pearson correlation of pairwise canonical variates in ten-fold cross validation (one-sided test against the alternative hypothesis that $r > 0$). Specifically, the dataset was evenly divided into ten subsets. sCCA was then trained and tested ten times, using a different fold as the test set and the remaining nine folds as the training set. The NPS and FC latent features transformed from sCCA on each test set were concatenated. Pearson correlation between the concatenated NPS and FC latent features was calculated for each canonical component.

Post-hoc analyses

With the identified neuropsychiatric subsyndromes, we further examined their associations with original FCs and NPS, respectively. We calculated the Pearson correlation coefficient to assess the association between the FC and subsyndrome-linked FC latent features. Since NPS scores were ordinal

© 2024 Zhao K et al. *JAMA Network Open*.

variables, we calculated the Spearman correlation coefficient to evaluate the association. Additionally, we examined the associations between other continuous phenotypic characteristics (such as age and MMSE) and subsyndromes-linked FC latent features using Pearson correlation and the associations between discrete phenotypic characteristics with subsyndrome-linked FC latent features using analysis of variance.

To investigate neural circuit abnormalities within each subtype, we conducted a comparative analysis of FC between dementia patients in each subtype and healthy controls using the Wilcoxon signed-rank test. We applied FDR to correct the p-values of the detected FC differences for each subtype. The Chi-square test (two tailed) identified associations between categorical phenotypes and dementia patients in each subtype. For ordinal phenotypes, we employed the Kruskal-Wallis analysis of variance to detect differences across dementia patients in each subtype. In cases where significant phenotypic differences were observed across all subtypes, we used Dunn's test to further examine the pairwise relationships with FDR correction. To examine longitudinal progression of cognitive abnormalities within each subtype, linear mixed-effect models were employed. Dependent variables included characteristic assessment scores at each study visit, and independent variables of fixed items included subtype label, time state, and the interaction of subtype label. We applied FDR to correct the p-values of interaction effects in all phenotypic items. Only the subjects with more than 95% chance assigned into the same cluster were used for the post-doc statistical analyses (n = 160).

To assess the longitudinal stability of FC latent features, we utilized the acquired sCCA model to extract FC latent features of follow-up fMRI data from 71 subjects, and compute Pearson correlations between baseline and follow-up FC latent features. The baseline FC latent features exhibited significant correlations with their longitudinal counterparts, indicating the longitudinal consistency of FC latent features (Figure S14). Moreover, to examine the influence of healthy controls on the identification of dementia subtypes, we applied K-means clustering solely to dementia patients. When clustering solely dementia patients, subtype 1 remained relatively stable, but the number of dementia within subtype 3 increased (Figure S15). This suggests that incorporating healthy controls into clustering provided a subtype 3 more representative of normal aging. In addition, we explored the impact of principal

© 2024 Zhao K et al. *JAMA Network Open*.

component selections of NPS on subtype identification using sCCA. Results showed that six or eight principal components decreased the average correlations between FC and NPS latent features compared to seven components (Figures S16A and D, Figures 2A, 3A). However, the contribution of FCs to the behavioral and anxiety subsyndrome-linked FC latent features and corresponding clustering results remained consistent using six to eight principal components (Figures S16B-C, E-I; S4A, B).

© 2024 Zhao K et al. *JAMA Network Open*.

Supplementary results

eFigure 1. Correlation between pairs of neuropsychiatric symptoms. Only the significant correlations (p<0.05) were texted in the figure.

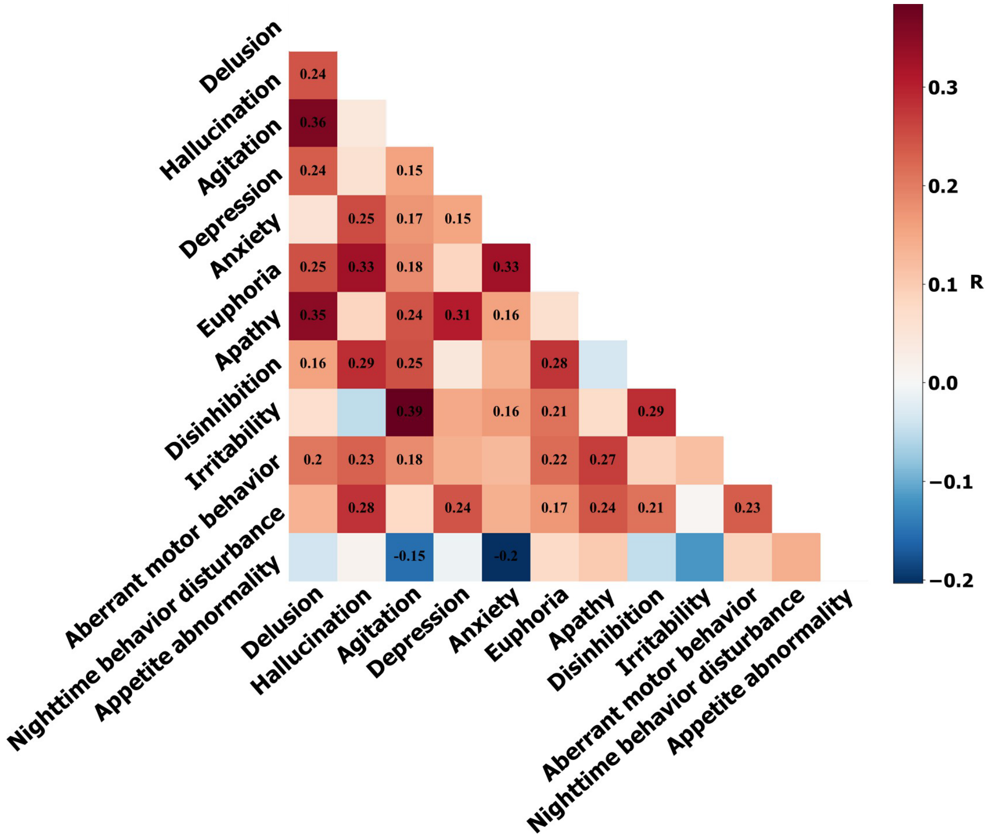

© 2024 Zhao K et al. *JAMA Network Open*.

eFigure 2. Hyperparameters selection in our study. A Variance explained by the principal components of NPS. **B** Average correlation of transformed pairwise canonical variates of ten-fold cross validation, with varying numbers of principal components of NPS and different L1 regularization parameters of connectivity features in sCCA. **C** Covariance explained by the canonical variates derived from sCCA between functional connectivity features and the first seven principal components of NPS.

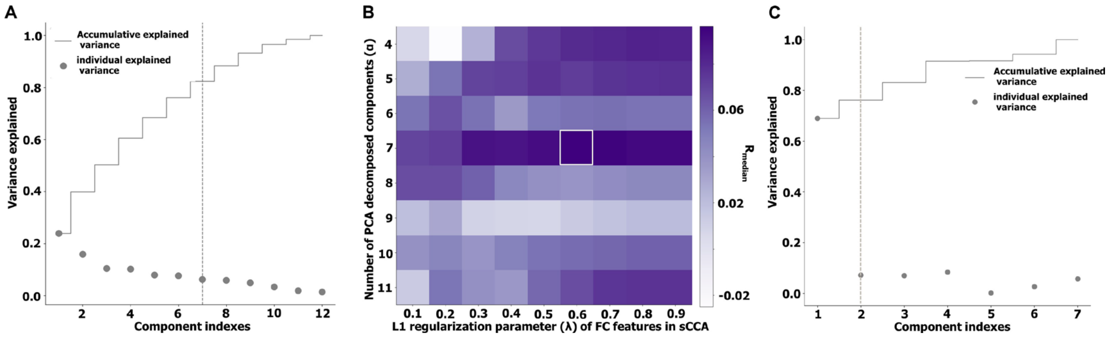

© 2024 Zhao K et al. *JAMA Network Open*.

eFigure 3. Top 50 (absolute) strongest correlations (FDR correction) between sCCA transformed FC latent features and FC features. **A**, Top 50 FCs correlated to behavioral subsyndrome-linked FC latent features. **B**, Top 50 FCs correlated to anxiety subsyndrome-linked FC latent features. The FCs are depicted in red or blue based on their positive or negative correlation, respectively. Node size indicated the node strength calculated from the summed FC strength of each ROI.

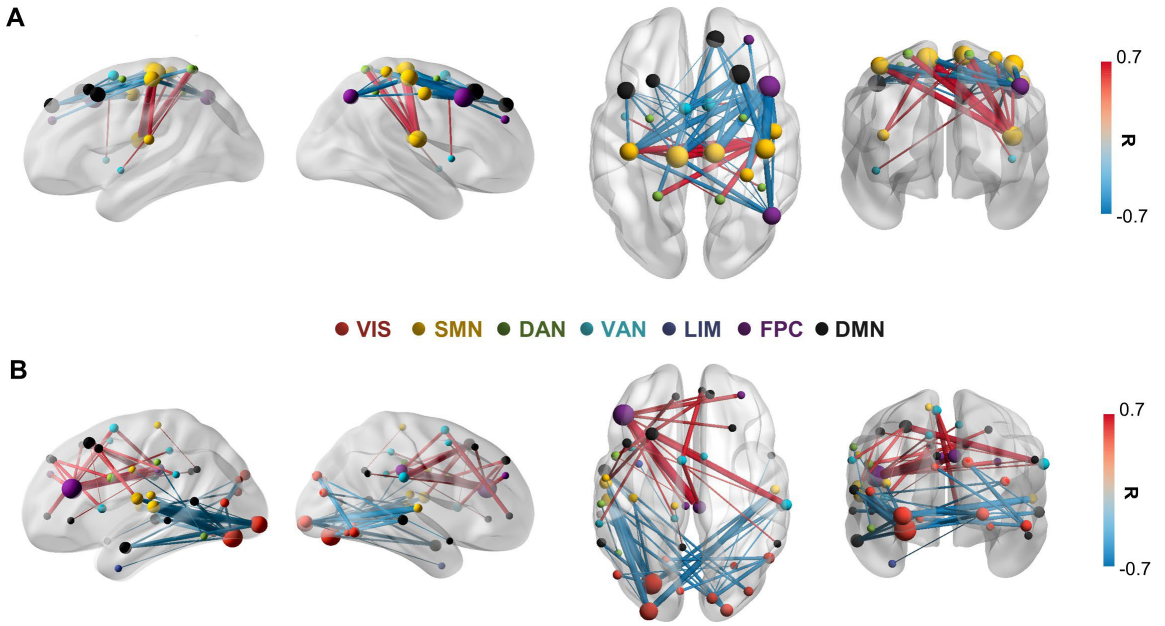

© 2024 Zhao K et al. *JAMA Network Open*.

eFigure 4. Contribution of FCs to the first and second sCCA transformed FC variates. A, B The significant correlations (after FDR correction, $p_{fdr} < 0.05$) between FCs and the first and second sCCA transformed FC variates. Red color indicates positive correlation and blue color indicates negative correlation. **C, D** The weights in sCCA transformation matrices of the first and second canonical variates. Red color indicates positive weight and blue color indicates negative weight. **E**, **F**, **G**, **H** All significant correlations shown in **A, B**, and weights of sCCA shown in **C, D** were grouped into contribution of Yeo's 7 networks, including visual network (VIS), somatomotor network (SMN), dorsal attention network (DAN), ventral attention network (VAN), limbic network (LIM), frontoparietal control network (FPC), default mode network (DMN) by summation of the absolute correlation or canonical coefficients. Deeper purple and thicker lines represent larger contributions.

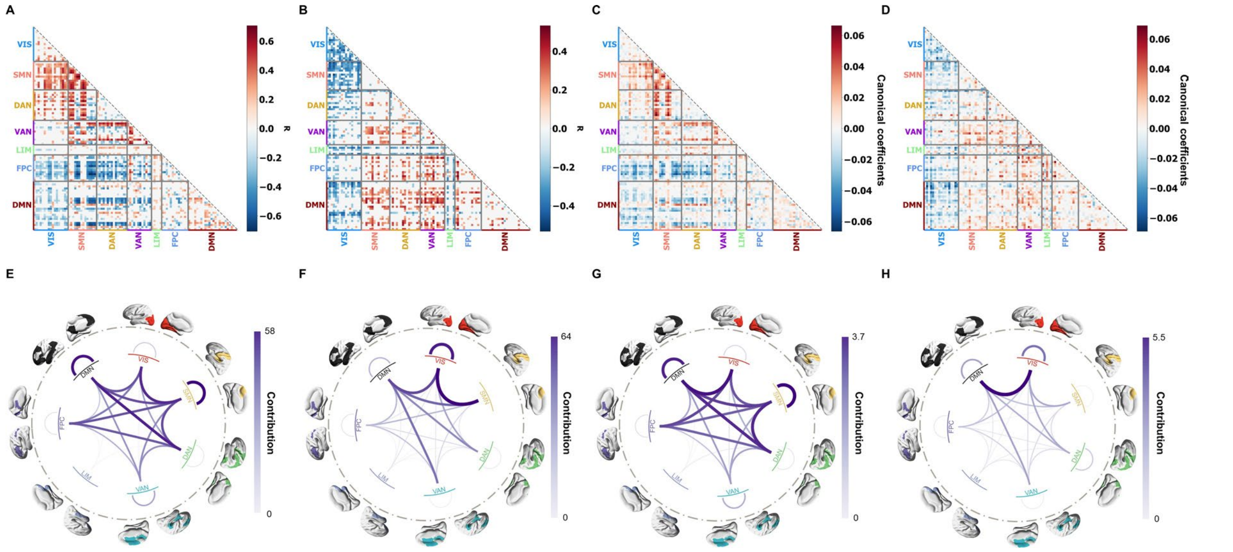

© 2024 Zhao K et al. *JAMA Network Open*.

eFigure 5. Tukey's honest significance (HSD) test results for the behavioral (A-F) and anxiety (G-L) subsyndromes across groups with different CDR and its subscale scores. All p values were FDR corrected. (NS: p > 0.05; *: p ≤ 0.05; **: p ≤ 0.01; ***: p ≤ 0.001; ****: p ≤ 0.0001). The HSD test was not conducted for the personal care subscale as it was a binary categorical variable.

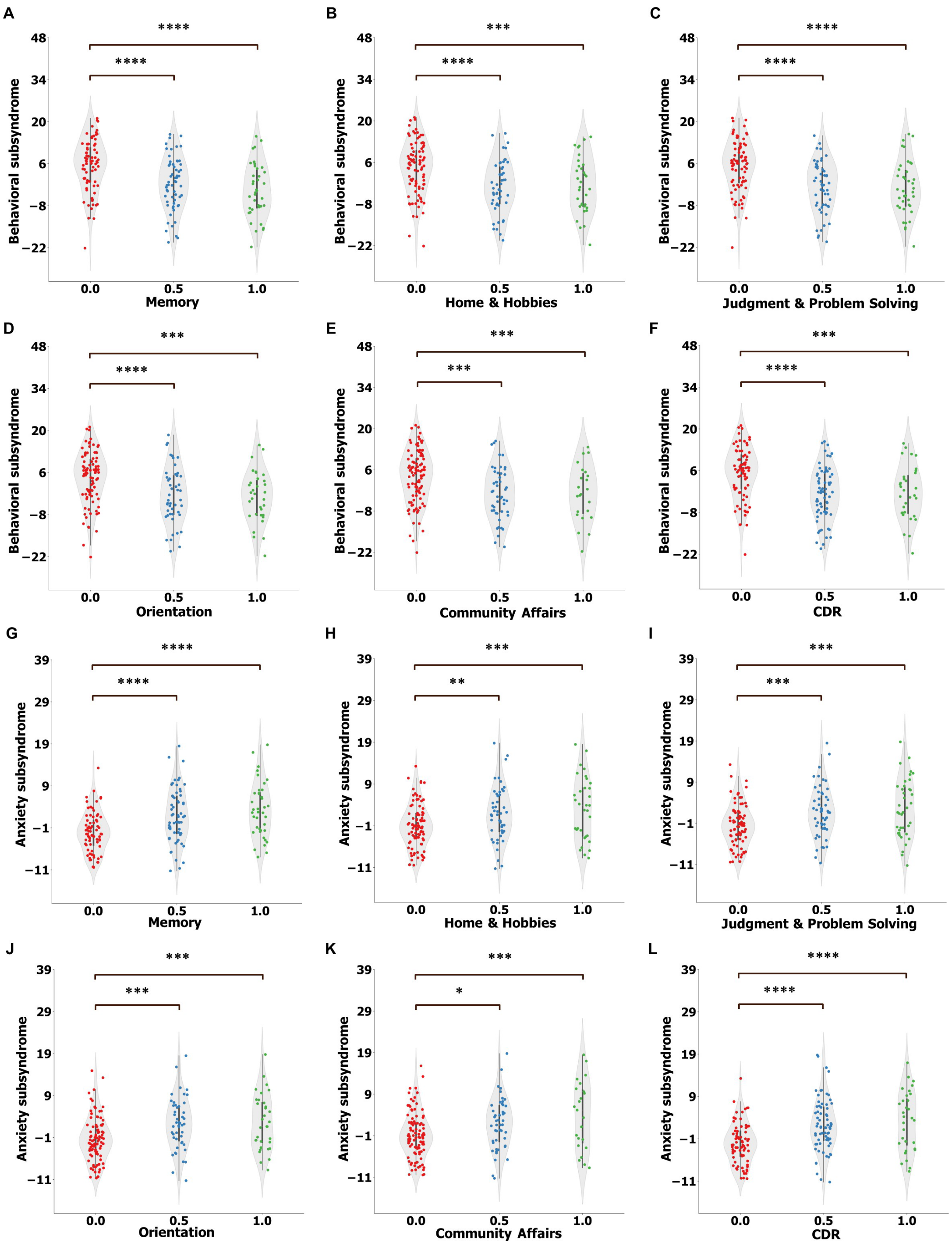

© 2024 Zhao K et al. *JAMA Network Open*.

eFigure 6. Tukey's honest significance (HSD) test of the first (A-E) and second (F-I) canonical variates across groups with different functional assessment items. Pairwise difference analysis using HSD was conducted only for the items that showed significant differences in canonical variates across all groups, as determined by Kruskal-Wallis analysis. All p values were FDR corrected. (NS: $p > 0.05$; *: $p \leq 0.05$; **: $p \leq 0.01$; ***: $p \leq 0.001$; ****: $p \leq 0.0001$).

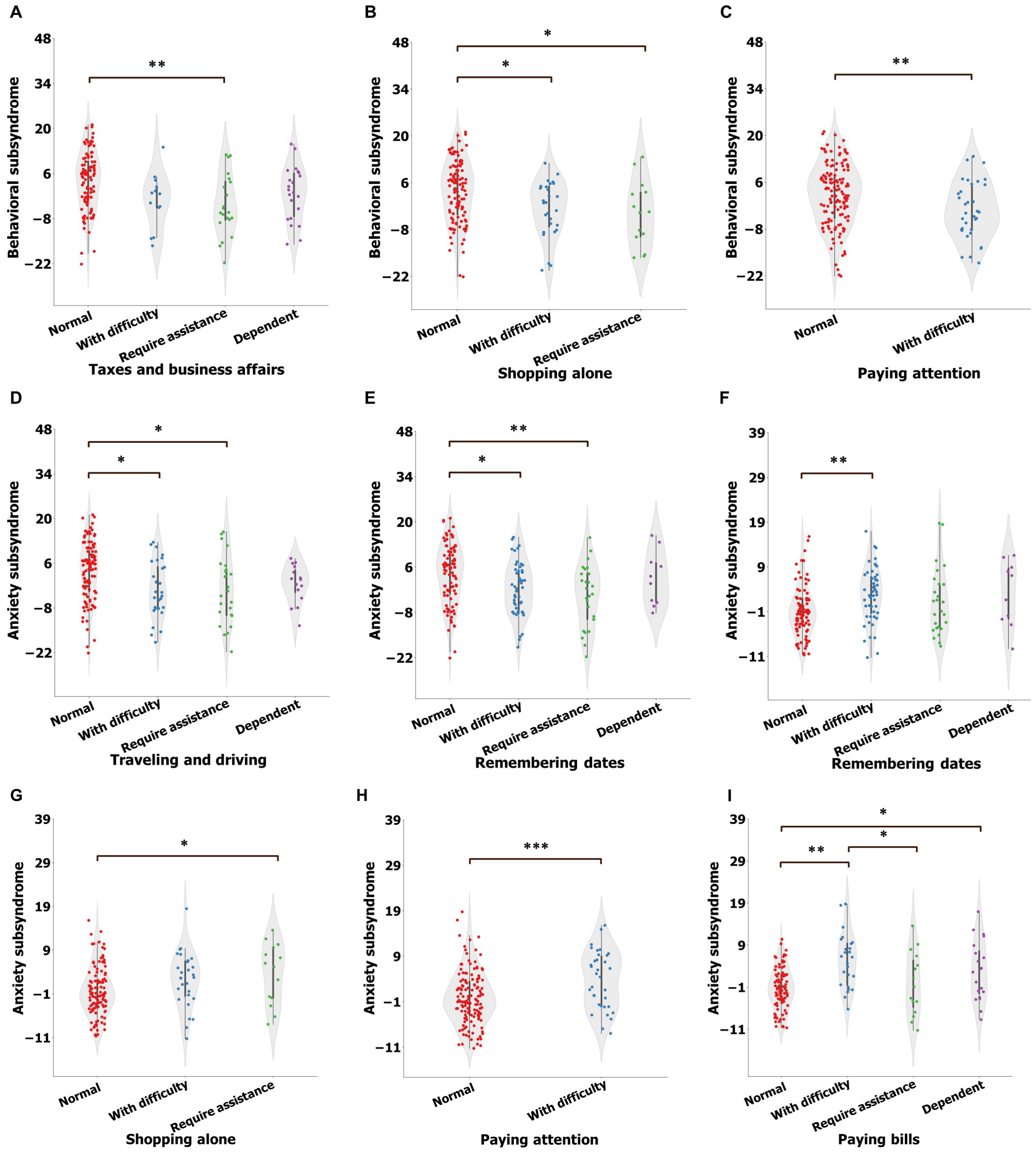

© 2024 Zhao K et al. *JAMA Network Open*.

eFigure 7. Cluster evaluation analyses of K-means clustering. We repeated K-means cluster 1000 times, with 90% random subsampling of all subjects. When cluster number (k) was 3, **A** the Calinski-Harabasz score and **B** Silhouette score, were maximized. **C** The stability coefficient, represented as the ratio of the same cluster label assigned to each subject across 1000 subsamplings, was calculated. The stability coefficients of different cluster numbers were plotted. **D** When k = 3, the stability coefficients of subjects in different subtypes were computed.

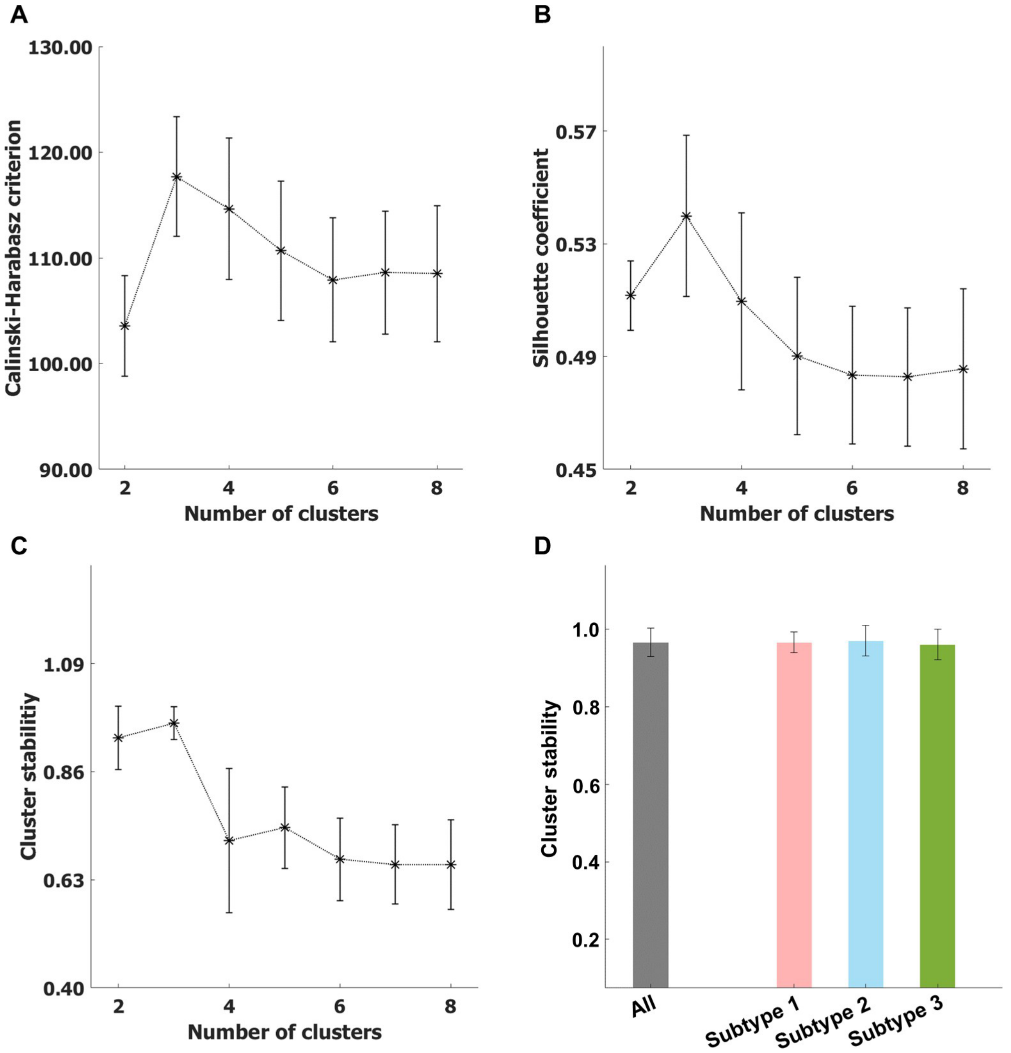

© 2024 Zhao K et al. *JAMA Network Open*.

eFigure 8. Cluster evaluation analyses of hierarchical clustering. A The visualization of hierarchical cluster result, when k = 3. Jaccard similarity was 1, compared to the subtype labels from K-means clustering. We then repeated hierarchical clustering 1000 times, with 90% random subsampling of all subjects. When cluster number (k) was 3, **B** the Calinski-Harabasz score and **C** Silhouette score, were maximized. **D** To assess stability of the cluster analysis when cluster number was 3, the stability coefficient of different subtypes was computed as the ratio of the same cluster label assigned to subjects in each subtype across the 1000 subsamplings.

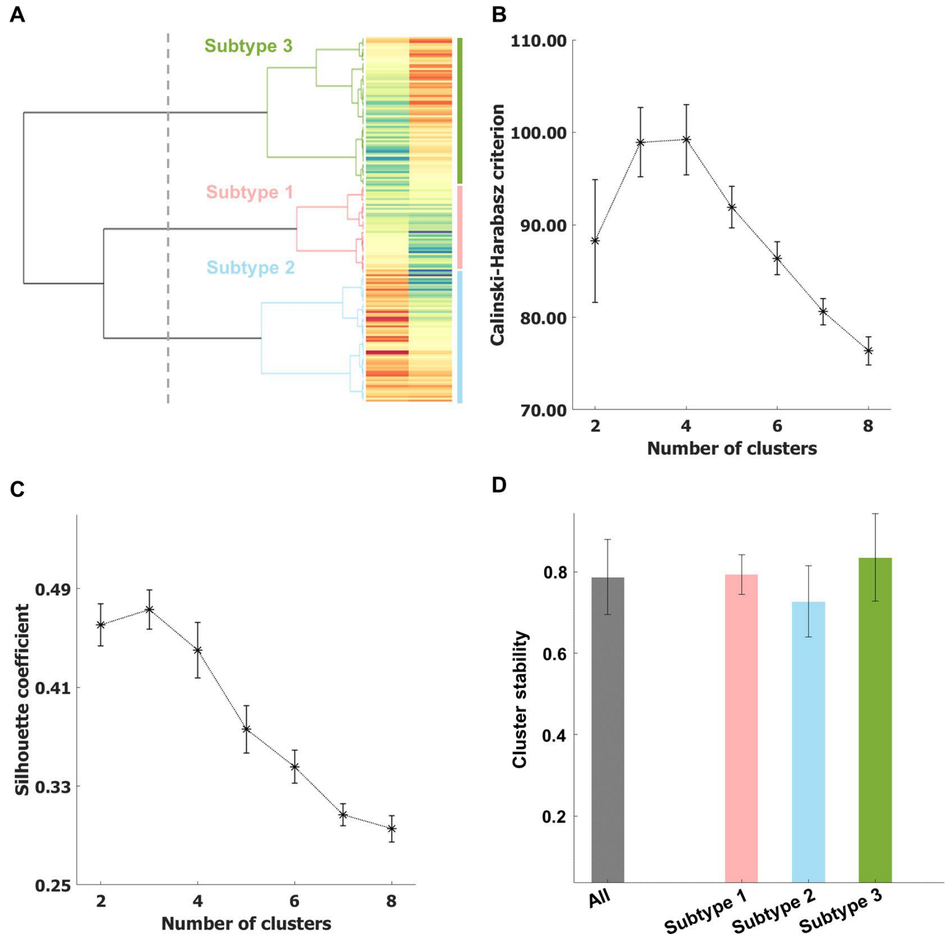

© 2024 Zhao K et al. *JAMA Network Open*.

eFigure 9. Comparing FCs of dementia in subtypes 1 and 2 with all subjects in healthy controls. The differences were detected using z score of the two-side Wilcoxon rank sum test and the significance of differences was corrected by FDR ($p_{fdr} < 0.05$). Hyperconnections were represented in red, indicating that the FCs of healthy controls were larger than those in dementia patients for a subtype. Hypoconnections are represented in blue, indicating that FCs of healthy controls were smaller than those in dementia patients for a subtype. Node size indicated the node strength calculated from the sum of the absolute w value of linked FC. **A, B** The z scores of top 50 (absolute) significant abnormal FCs of dementia in subtypes 1 and 2. **C, D** ROI-level FCs differences of dementia in subtypes 1 and 2. **E, F** Network-level FCs differences of dementia in subtypes 1 and 2. In the chord plot, deeper purple and thicker lines represent larger differences summed from the absolute z scores from each ROI.

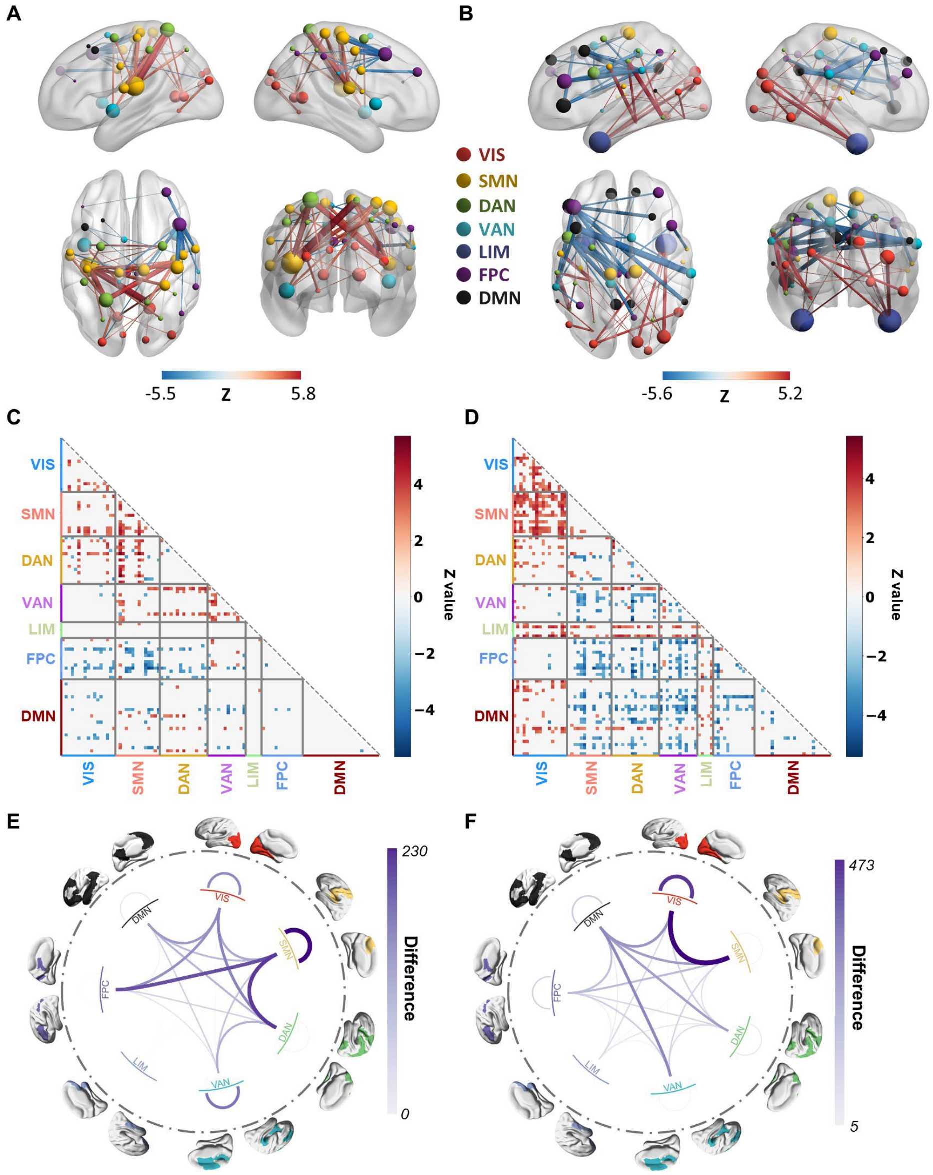

© 2024 Zhao K et al. *JAMA Network Open*.

eFigure 10. Dunn's multiple comparison results of all ordinal clinical measurements, which were significantly different across three dementia subtypes. All p values were FDR corrected. (NS: p > 0.05; *: p ≤ 0.05; **: p ≤ 0.01; ***: p ≤ 0.001; ****: p ≤ 0.0001).

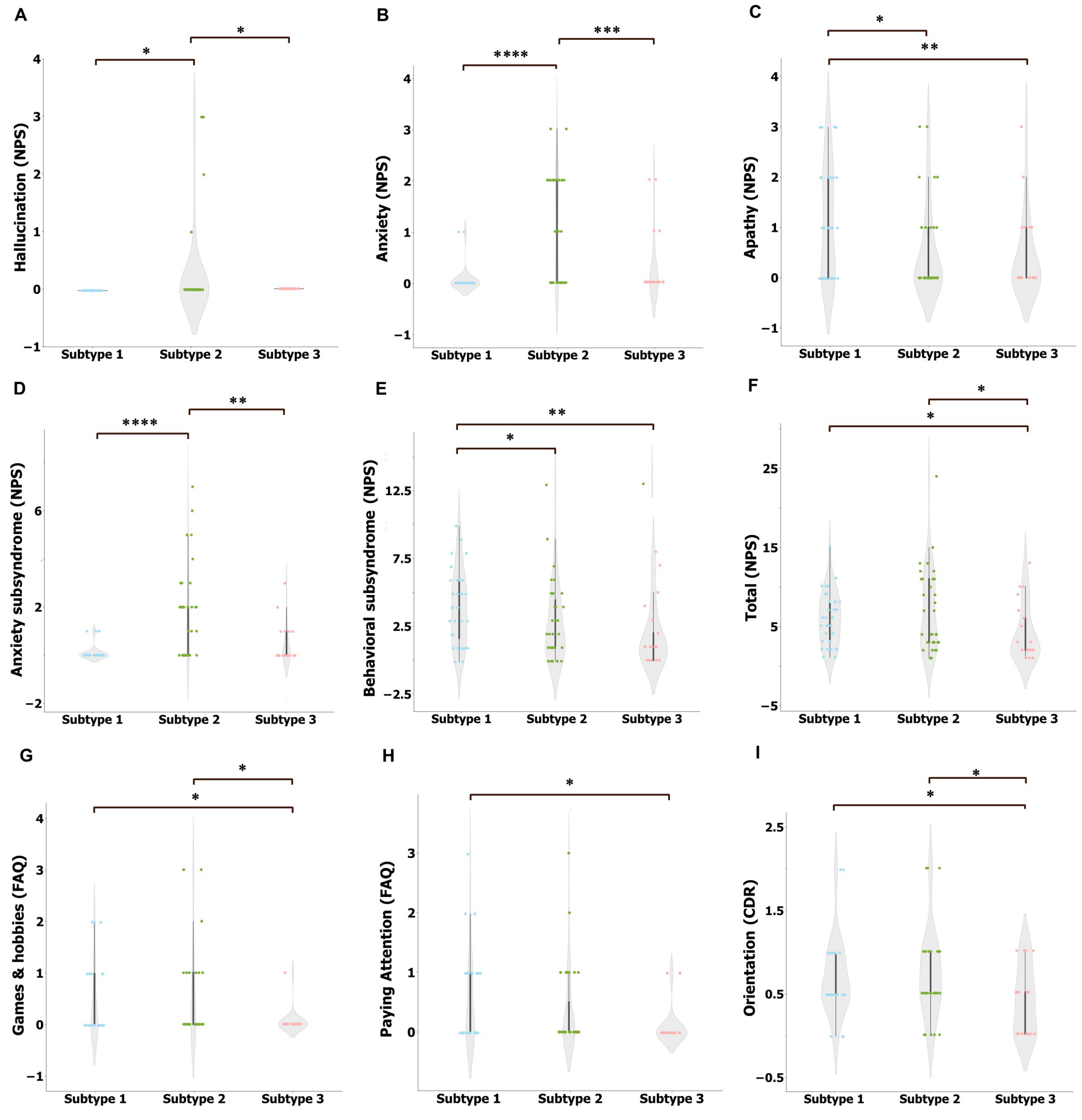

© 2024 Zhao K et al. *JAMA Network Open*.

eFigure 11. Differences in longitudinal change of various clinical scores among dementia in subtypes. Linear mixed effect models were employed to examine the difference in the longitudinal progression of clinical scores of dementia across subtypes. Only the clinical scores that exhibited significant differences in their longitudinal trajectories among the three dementia subtypes are presented.

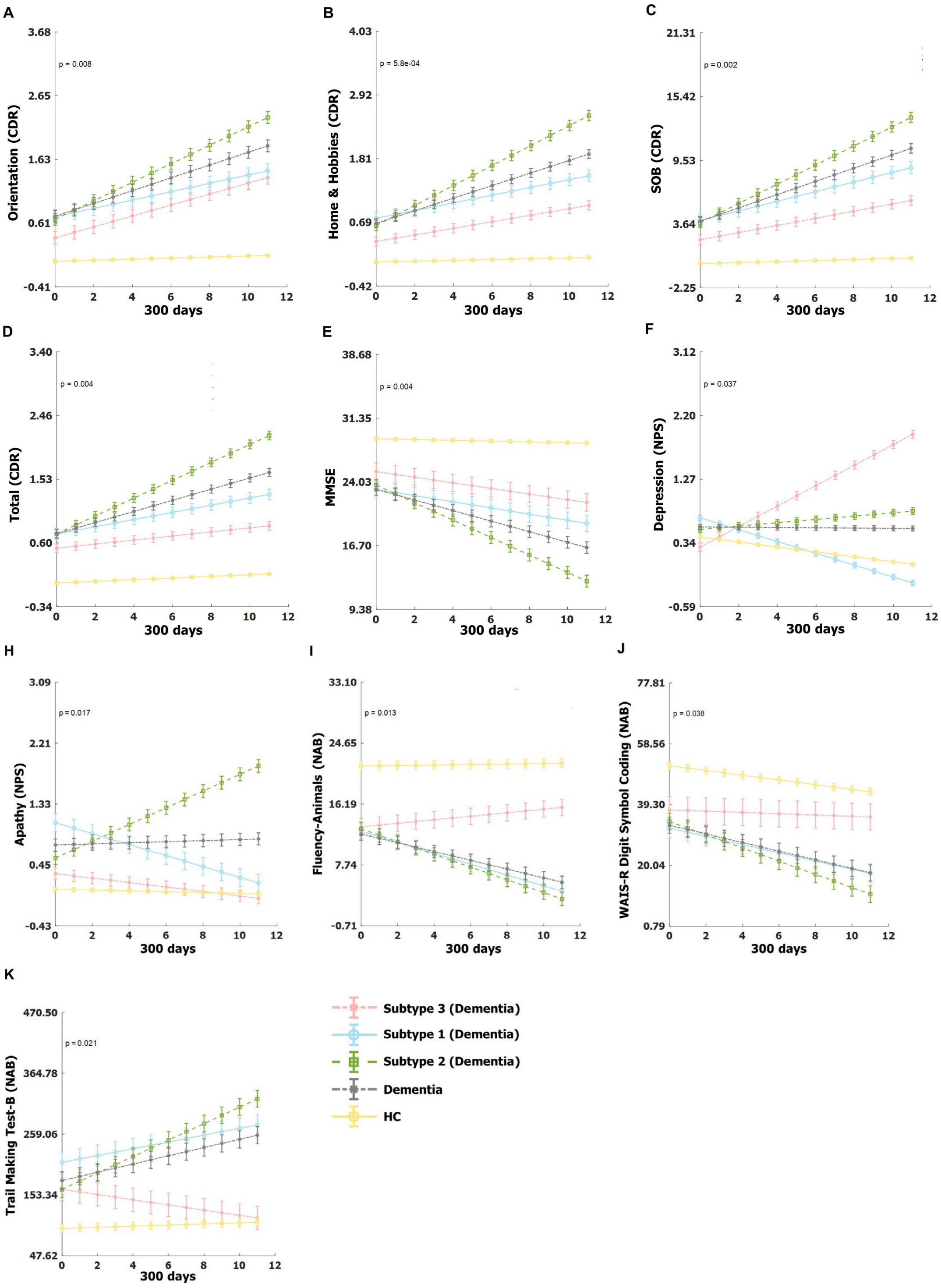

© 2024 Zhao K et al. *JAMA Network Open*.

eFigure 12. Clustering on NPS scores only. A Calinski-Harabasz score, **B** Silhouette score and **C** stability coefficient of different cluster numbers. **D** Confusion matrix comparing subtype labels obtained from clustering NPS scores only (x-axis) to clustering based on the NPS-linked FC latent scores (y-axis). The accuracy is 0.41. **E** Differences of FCs in dementia of subtype 1, compared to all healthy controls. **F** Difference of FCs in dementia of subtype 2, compared to all healthy controls. The differences were detected using the Wilcoxon rank sum test, and the significance of difference was corrected by FDR.

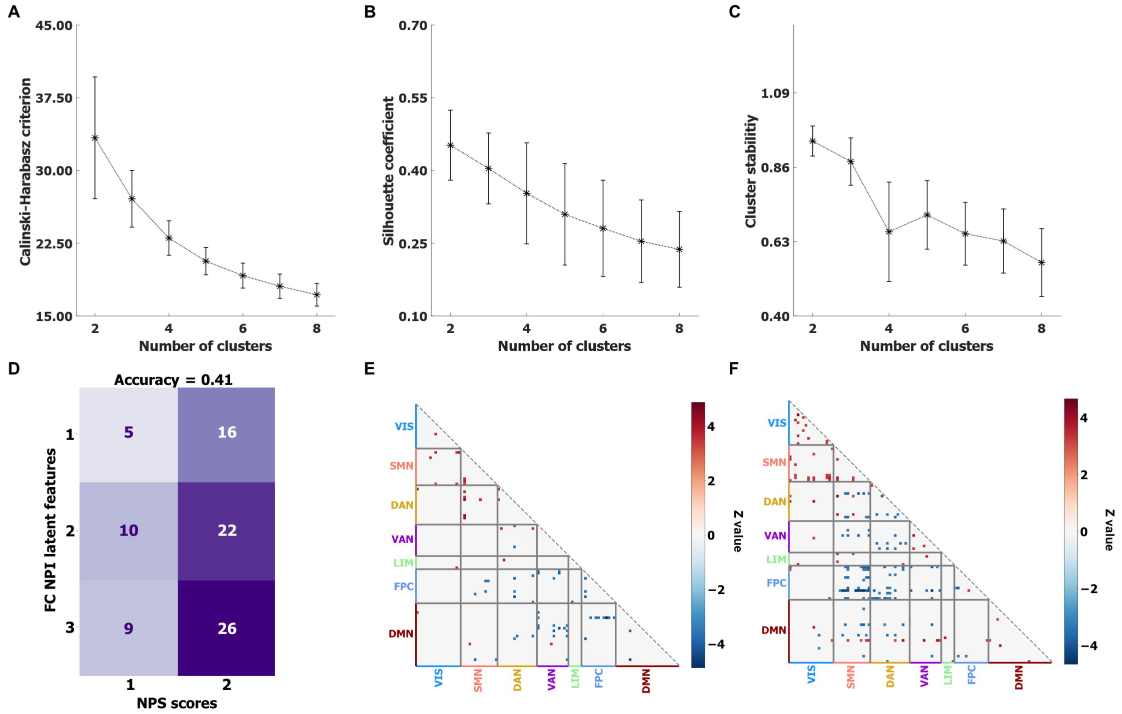

© 2024 Zhao K et al. *JAMA Network Open*.

eFigure 13. Consistency in longitudinal changes of NPS subsyndromes-linked FC latent features. We applied the obtained CCA model to extract the FC latent features transformed from the follow-up fMRI of subjects. **A, B** The scatter plot of the behavioral, anxiety subsyndromes-linked FC latent features obtained between baseline and follow-up fMRI of same subjects.

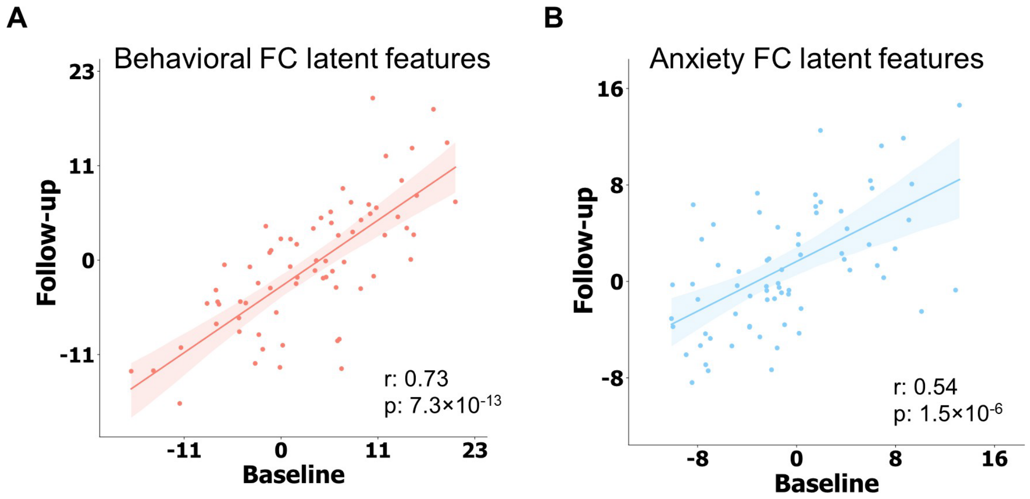

© 2024 Zhao K et al. *JAMA Network Open*.

eFigure 14. Association between the subtypes clustering on subjects including and excluding healthy controls (HCs). The left panel is a scatter plot of clustering results using dementia patients only. The right panel shows counts of patients assigned into same subtypes or not, clustering including or excluding HCs. Subtype (all): subtypes obtained by clustering all subjects including HCs; Subtype (only dementia): subtypes obtained by clustering only dementia patients. We used Jacaard score to measure the similarity of dementia in each subtype clustering between including healthy control and excluding healthy control. The Jaccard scores were 0.89, 0.63, 0.51 for subtypes 1, 2, 3, respectively.

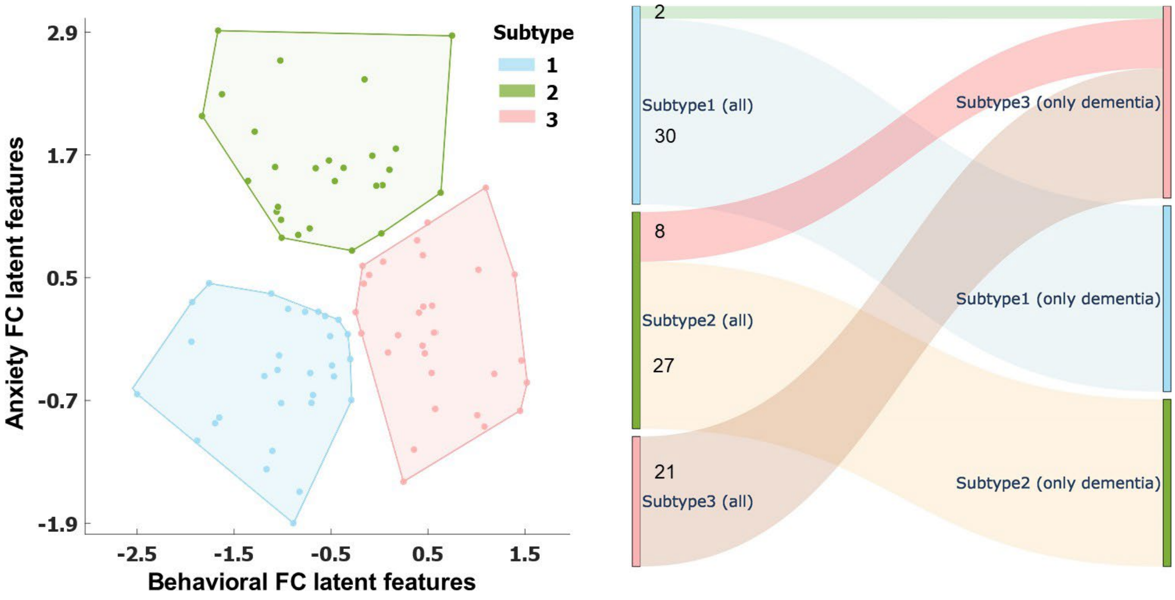

© 2024 Zhao K et al. *JAMA Network Open*.

eFigure 15. Principal components influence to identification of subtypes. We selected 6 and 8 principal components of NPS and used those components with FC to identify the first and second canonical latent features. A-C The results from 6 components of NPS. **A** The scatter plots of the first (behavioral) and second (anxiety) canonical latent features in test set. **B** Correlation between FCs and the behavioral subsyndrome-linked FC latent features. **C** Correlation between FCs and the anxiety subsyndrome-linked FC latent features. **D-F** The results from 8 components of NPS. **D** The scatter plots of the first (behavioral) and second (anxiety) canonical latent features in test set. **E** Correlation between FCs and the behavioral subsyndrome-linked FC latent features. **F** Correlation between FCs and the anxiety subsyndrome-linked FC latent features. **G, H** The subtyping results when k=3, applying K-means to two subsyndrome-linked FC latent features transformed from 6 and 8 principal components of NPS. **I** Counts of subjects assigned into same subtypes using different NPS principal components.

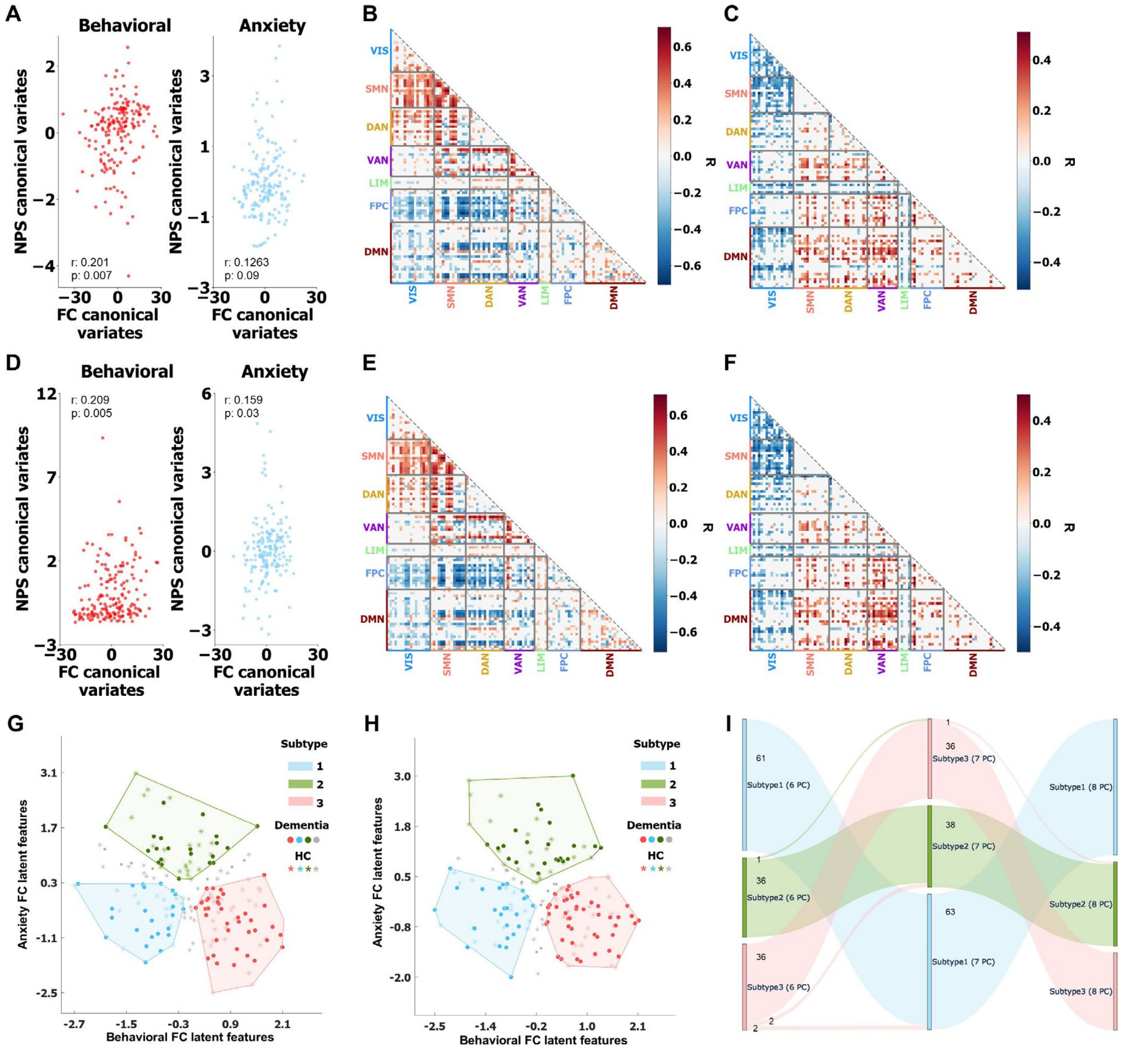

© 2024 Zhao K et al. *JAMA Network Open*.

eFigure 16. Replicability verified in the replication dataset. **A** Correlation between FC and NPS latent features, transformed from obtained PCA and sCCA models (behavioral subsymdrome: r = 0.24, p = 0.0009; anxiety subsymdrome: r = 0.20, p = 0.005). **B** Scatter plot of three subtypes of subjects along the dimension of anxiety subsyndrome and behavioral subsyndrome. **C, D** Comparing FCs of dementia in subtypes 1 and 2 with all subjects in healthy controls, using Wilcoxon rank sum test ($p_{fdr} < 0.05$). The upper right chord plots were the network-level difference. **E-H** Dunn's multiple comparison results of all ordinal clinical measurements, which were significantly different across three dementia subtypes. All p values were FDR corrected. (NS: p > 0.05; *: p ≤ 0.05; **: p ≤ 0.01; ***: p ≤ 0.001; ****: p ≤ 0.0001).

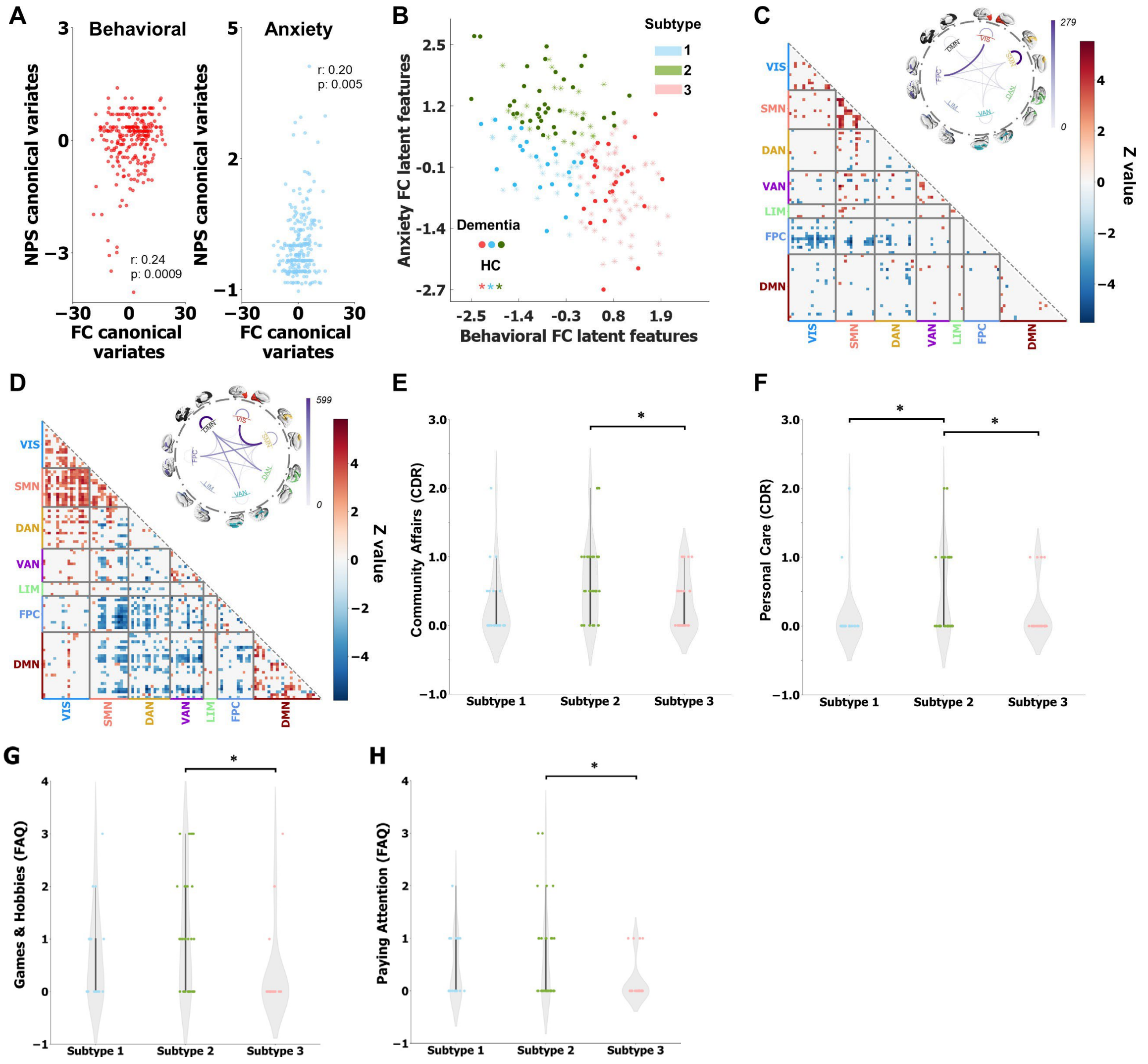

© 2024 Zhao K et al. *JAMA Network Open*.

eTable 1. Summary the correlation between the third to seventh sCCA transformed connectivity latent scores and NPS latent scores. All p values were FDR corrected.

Canonical variates index	r	p	$p_{permute}$
3rd	-0.174	0.020	0.974
4th	-0.013	0.859	0.583
5th	0.072	0.337	0.459
6th	0.161	0.033	0.201
7th	-0.006	0.941	0.583

© 2024 Zhao K et al. *JAMA Network Open*.

eTable 2. Characteristic information of subjects from the OASIS-3 and ADNI cohorts used in this study. The difference in gender between dementia and healthy controls was assessed using the Chi-squared test while difference in age was assessed through two-sample t-test or Wilcoxon signed-rank test.

**OASIS-3 Characteristics**	**HC**		**Dementia**		**Statistic values**	
	n	%	n	%	$\chi 2$	p
Gender					0.55	0.46
Male	71	42	76	47		
Female	97	58	86	53		
	mean	std	mean	std	t	p
Age (baseline)	69	10	75	8	-5.8	2.1×10^{-8}
**ADNI Characteristics**	**HC**		**Dementia**		**Statistic values**	
	n	%	n	%	$\chi 2$	p
Gender						
Male	4	40	13	45	0	1
Female	6	60	16	55		
	median	interquartile ranges	median	interquartile ranges	z(w)	p
Age (baseline)	74	4	73	7	0.5	0.64

© 2024 Zhao K et al. *JAMA Network Open*.

eTable 3. Characteristic information of subjects from the discovery dataset. The difference in gender between dementia and healthy controls was assessed using the Chi-squared test while difference in age was assessed through Wilcoxon signed-rank test.

Characteristic variables	HC		Dementia		Statistic values	
	n	%	n	%	$\chi 2$	p
Gender					3.9	0.05
Male	35	47	64	63		
Female	40	53	38	37		
	median	interquartile ranges	median	interquartile ranges	z(w)	p
Age (baseline)	68	10	74	9	-5.4	8.1×10^{-8}

© 2024 Zhao K et al. *JAMA Network Open*.

eTable 4. Characteristic information of subjects from replication dataset. The difference in gender between dementia and healthy controls was assessed using the Chi-squared test while differences in age were assessed through Wilcoxon signed-rank test.

Characteristic variables	HC		Dementia		Statistic values	
	n	%	n	%	$\chi2$	p
Gender					1.2	0.27
Male	40	38	26	30		
Female	65	62	62	70		
	median	interquartile ranges	median	interquartile ranges	z(w)	p
Age (baseline)	71	12	77	9	-4.0	6.9×10^{-5}

© 2024 Zhao K et al. *JAMA Network Open*.

eTable 5. Relationship between FC latent scores and the continuous clinical measurements was measured by using Pearson correlation. All p values were FDR corrected. (NS: $p > 0.05$; *: $p \leq 0.05$; **: $p \leq 0.01$; ***: $p \leq 0.001$; ****: $p \leq 0.0001$)

	Behavioral subsyndrome		Anxiety subsyndrome	
	r	p_{fdr}	r	p_{fdr}
**MMSE**	**0.309**	****	**-0.304**	****
**Digit Span-Forward (NAB)**	0.135	NS	0.002	NS
**Digit Span-Backward (NAB)**	0.081	NS	-0.087	NS
**Fluency-Animals (NAB)**	0.129	NS	**-0.232**	**
**Fluency-Vegetable (NAB)**	**0.243**	**	**-0.255**	***
**Trail Making Test-A (NAB)**	**-0.190**	*	**0.222**	**
**Trail Making Test-B (NAB)**	-0.189	NS	**0.221**	*
**WAIS-R Digit Symbol Coding (NAB)**	**0.224**	**	**-0.262**	***
**Logical Memory Immediate (NAB)**	**0.201**	*	**-0.232**	**
**Logical Memory Delayed (NAB)**	0.159	NS	**-0.226**	**
**Boston (NAB)**	**0.193**	*	**-0.168**	*

© 2024 Zhao K et al. *JAMA Network Open*.

eTable 6. Association between FC latent scores and the scores of various categorical clinical measurements and biomarkers. Statistical comparisons were performed using ANOVA and two-sample t-test. All p values were FDR corrected. (NS: $p > 0.05$; *: $p \leq 0.05$; **: $p \leq 0.01$; ***: $p \leq 0.001$; ****: $p \leq 0.0001$)

	**Behavioral subsyndrome**		**Anxiety subsyndrome**	
	**f/t**	**p_{fdr}**	**f/t**	**p_{fdr}**
**Personal Care (CDR)**	2.26	NS	2.08	NS
**Memory (CDR)**	**13.40**	****	**13.50**	****
**Home & hobbies (CDR)**	**10.20**	****	**7.38**	***
**Judgment & problem solving (CDR)**	**14.70**	****	**7.94**	***
**Orientation (CDR)**	**9.74**	****	**8.89**	***
**Community Affairs (CDR)**	**7.64**	***	**7.44**	***
**Total (CDR)**	**12.70**	****	**13.50**	****
**SOB (CDR)**	**-0.274**	***	**0.345**	****
**APOE**	1.56	NS	2.18	NS
**Diagnosis Label**	**4.95**	****	**-4.41**	****
**Paying Bills (FAQ)**	2.33	NS	**10.2**	****
**Taxes and Business Affairs (FAQ)**	**5.27**	**	2.67	NS
**Shopping Alone (FAQ)**	**5.88**	**	**5.20**	*
**Games and Hobbies (FAQ)**	4.65	NS	1.23	NS
**Using Stove (FAQ)**	2.31	NS	1.42	NS
**Preparing a Balanced Meal (FAQ)**	1.05	NS	2.07	NS
**Current Events (FAQ)**	1.82	NS	2.89	NS
**Paying Attention (FAQ)**	**6.71**	*	**11.8**	**
**Remembering Dates (FAQ)**	**4.70**	**	**4.60**	**
**Traveling and Driving (FAQ)**	**4.96**	**	2.83	NS

© 2024 Zhao K et al. *JAMA Network Open*.

eTable 7. Associations between FC latent scores and demographic information. Two-sample t-test and Pearson correlation were applied. All p values were FDR corrected. (NS: $p > 0.05$; *: $p \leq 0.05$; **: $p \leq 0.01$; ***: $p \leq 0.001$; ****: $p \leq 0.0001$)

	**Behavioral subsyndrome**		**Anxiety subsyndrome**	
	**r/t**	$\mathbf{p_{fdr}}$	**r/t**	$\mathbf{p_{fdr}}$
**Age**	**-0.351**	****	**0.186**	*
**Sex**	0.893	NS	**10.90**	**
**Education**	-0.087	NS	0.036	NS

© 2024 Zhao K et al. *JAMA Network Open*.

eTable 8. Differences of various clinical measurements and demographic information across dementia in three subtypes defined from FC-linked latent features. Kruskal–Wallis ANOVA and Chi-square were applied in statistic comparisons of ordinal and categorical variables respectively, unless corresponding variables of each group were equal. (NS: $p > 0.05$; *: $p \leq 0.05$; **: $p \leq 0.01$; ***: $p \leq 0.001$; ****: $p \leq 0.0001$)

Variables	f/χ^2	p
**Age**	3.69	NS
**Sex**	4.06	NS
**Education**	0.46	NS
**Delusion (NPS)**	3.77	NS
**Hallucination (NPS)**	**7.93**	*
**Agitation (NPS)**	0.48	NS
**Depression (NPS)**	5.74	NS
**Anxiety (NPS)**	**26.4**	****
**Euphoria (NPS)**	4.66	NS
**Apathy (NPS)**	**8.63**	**
**Disinhibition (NPS)**	3.25	NS
**Irritability (NPS)**	1.04	NS
**Aberrant Motor Behavior (NPS)**	1.21	NS
**Nighttime Behavior Disturbances (NPS)**	4.55	NS
**Appetite Abnormalities (NPS)**	1.11	NS
**Behavioral subsyndrome (NPS)**	5.43	NS
**Anxiety subsyndrome (NPS)**	**10.6**	**
**Total (NPS)**	**25.8**	****
**APOE**	6.88	NS
**Personal Care (CDR)**	2.63	NS
**Memory (CDR)**	5.45	NS
**Home & hobbies (CDR)**	3.30	NS
**Judgment & problem solving (CDR)**	3.08	NS
**Orientation (CDR)**	**6.62**	*
**Community Affairs (CDR)**	3.23	NS
**Total (CDR)**	2.88	NS

© 2024 Zhao K et al. *JAMA Network Open*.

**SOB (CDR)**	5.83	NS
**MMSE**	3.51	NS
**Digit Span-Forward (NAB)**	3.20	NS
**Digit Span-Backward (NAB)**	1.73	NS
**Fluency-Animals (NAB)**	1.30	NS
**Fluency-Vegetable (NAB)**	0.93	NS
**Trail Making Test-A (NAB)**	3.70	NS
**Trail Making Test-B (NAB)**	1.73	NS
**WAIS-R Digit Symbol Coding (NAB)**	2.40	NS
**Logical Memory Immediate (NAB)**	2.02	NS
**Logical Memory Delayed (NAB)**	0.19	NS
**Boston (NAB)**	0.18	NS
**Paying Bills (FAQ)**	5.74	NS
**Taxes and Business Affairs (FAQ)**	1.44	NS
**Shopping Alone (FAQ)**	5.29	NS
**Games and Hobbies (FAQ)**	**6.52**	*
**Using Stove (FAQ)**	0.29	NS
**Preparing a Balanced Meal (FAQ)**	4.01	NS
**Current Events (FAQ)**	2.95	NS
**Paying Attention (FAQ)**	**6.36**	*
**Remembering Dates (FAQ)**	4.24	NS
**Traveling and Driving (FAQ)**	5.43	NS

eTable 9. Difference of various clinical measurements and demographic information across dementia in subtypes defined from NPS. Kruskal–Wallis ANOVA and Chi-square were applied in the statistic comparisons of ordinal and categorical variables respectively, unless corresponding variables of each group were equal. (NS: $p > 0.05$; *: $p \le 0.05$; **: $p \le 0.01$; ***: $p \le 0.001$; ****: $p \le 0.0001$)

Variables	f/χ^2	p
**Age**	0.09	NS
**Sex**	5.89	NS
**Education**	2.07	NS
**Delusion (NPS)**	3.39	NS
**Hallucination (NPS)**	1.43	NS
**Agitation (NPS)**	3.87	NS
**Depression (NPS)**	0.89	NS
**Anxiety (NPS)**	4.97	NS
**Euphoria (NPS)**	5.90	NS
**Apathy (NPS)**	5.24	NS
**Disinhibition (NPS)**	0.32	NS
**Irritability (NPS)**	1.31	NS
**Aberrant Motor Behavior (NPS)**	5.96	NS
**Nighttime Behavior Disturbances (NPS)**	1.50	NS
**Appetite Abnormalities (NPS)**	2.32	NS
**Behavioral subsyndrome (NPS)**	3.07	NS
**Anxiety subsyndrome (NPS)**	**6.08**	*
**Total (NPS)**	3.46	NS
**APOE**	3.89	NS
**Personal Care (CDR)**	2.40	NS
**Memory (CDR)**	1.36	NS
**Home & hobbies (CDR)**	1.36	NS
**Judgment & problem solving (CDR)**	0.96	NS
**Orientation (CDR)**	1.72	NS
**Community Affairs (CDR)**	2.26	NS
**Total (CDR)**	1.37	NS
**SOB (CDR)**	1.62	NS

© 2024 Zhao K et al. *JAMA Network Open.*

**MMSE**	0.14	NS
**Digit Span-Forward (NAB)**	3.63	NS
**Digit Span-Backward (NAB)**	1.41	NS
**Fluency-Animals (NAB)**	0.38	NS
**Fluency-Vegetable (NAB)**	1.26	NS
**Trail Making Test-A (NAB)**	0.13	NS
**Trail Making Test-B (NAB)**	1.06	NS
**WAIS-R Digit Symbol Coding (NAB)**	0.97	NS
**Logical Memory Immediate (NAB)**	1.86	NS
**Logical Memory Delayed (NAB)**	3.23	NS
**Boston (NAB)**	0.82	NS
**Paying Bills (FAQ)**	**12.7**	**
**Taxes and Business Affairs (FAQ)**	**12.0**	**
**Shopping Alone (FAQ)**	**13.2**	**
**Games and Hobbies (FAQ)**	**14.8**	***
**Using Stove (FAQ)**	**8.66**	*
**Preparing a Balanced Meal (FAQ)**	5.46	NS
**Current Events (FAQ)**	**11.0**	**
**Paying Attention (FAQ)**	**12.7**	**
**Remembering Dates (FAQ)**	**16.5**	***
**Traveling and Driving (FAQ)**	**16.5**	***

© 2024 Zhao K et al. *JAMA Network Open*.

eTable 10. Differences of various clinical measurements and demographic information across dementia in three subtypes defined from FC-linked latent features in the replication dataset. Kruskal–Wallis ANOVA and Chi-square were applied in statistic comparisons of ordinal and categorical variables respectively, unless corresponding variables of each group were equal. (NS: p > 0.05; *: p ≤ 0.05; **: p ≤ 0.01; ***: p ≤ 0.001; ****: p ≤ 0.0001)

Variables	f/χ^2	p
**Age**	3.34	NS
**Sex**	3.96	NS
**Delusion (NPS)**	1.44	NS
**Hallucination (NPS)**	0.66	NS
**Agitation (NPS)**	0.71	NS
**Depression (NPS)**	1.37	NS
**Anxiety (NPS)**	2.94	NS
**Euphoria (NPS)**	0.26	NS
**Apathy (NPS)**	0.60	NS
**Disinhibition (NPS)**	2.11	NS
**Irritability (NPS)**	0.96	NS
**Aberrant Motor Behavior (NPS)**	0.74	NS
**Nighttime Behavior Disturbances (NPS)**	1.19	NS
**Appetite Abnormalities (NPS)**	2.32	NS
**Behavioral subsyndrome (NPS)**	1.76	NS
**Anxiety subsyndrome (NPS)**	1.41	NS
**Total (NPS)**	1.94	NS
**Personal Care (CDR)**	**7.67**	*
**Memory (CDR)**	4.42	NS
**Home & hobbies (CDR)**	2.21	NS
**Judgment & problem solving (CDR)**	5.59	NS
**Orientation (CDR)**	3.16	NS
**Community Affairs (CDR)**	**7.91**	*
**Total (CDR)**	5.18	NS
**SOB (CDR)**	2.90	NS
**MMSE**	0.87	NS

© 2024 Zhao K et al. *JAMA Network Open*.

**Paying Bills (FAQ)**	1.54	NS
**Taxes and Business Affairs (FAQ)**	0.73	NS
**Shopping Alone (FAQ)**	4.03	NS
**Games and Hobbies (FAQ)**	**8.30**	*****
**Using Stove (FAQ)**	2.08	NS
**Preparing a Balanced Meal (FAQ)**	1.30	NS
**Current Events (FAQ)**	1.01	NS
**Paying Attention (FAQ)**	**6.29**	*****
**Remembering Dates (FAQ)**	**6.20**	*****
**Traveling and Driving (FAQ)**	3.93	NS

© 2024 Zhao K et al. *JAMA Network Open*.

Supplementary References

1. Morris JC. The Clinical Dementia Rating (CDR): current version and scoring rules. *Neurology.* 1993;43(11):2412-2414.
2. Tombaugh TN, McIntyre NJ. The mini-mental state examination: a comprehensive review. *J Am Geriatr Soc.* 1992;40(9):922-935.
3. Pfeffer RI, Kurosaki TT, Harrah Jr C, Chance JM, Filos S. Measurement of functional activities in older adults in the community. *Journal of gerontology.* 1982;37(3):323-329.
4. Morris JC, Weintraub S, Chui HC, et al. The Uniform Data Set (UDS): clinical and cognitive variables and descriptive data from Alzheimer Disease Centers. *Alzheimer Disease & Associated Disorders.* 2006;20(4):210-216.
5. Wechsler D. Manual for the Wechsler adult intelligence scale. 1955.
6. Morris JC, Heyman A, Mohs RC, et al. The consortium to establish a registry for Alzheimer's disease (CERAD): I. Clinical and neuropsychological assessment of Alzheimer's disease. *Neurology.* 1989.
7. Dikmen SS, Heaton RK, Grant I, Temkin NR. Test–retest reliability and practice effects of expanded Halstead–Reitan Neuropsychological Test Battery. *Journal of the International Neuropsychological Society.* 1999;5(4):346-356.
8. Kaplan E, Goodglass H, Weintraub S. Boston naming test. 2001.
9. ADNI2 Procedures Manual. *https://adniloniuscedu/wp-content/uploads/2008/07/adni2-procedures-manualpdf.*
10. Esteban O, Markiewicz CJ, Blair RW, et al. fMRIPrep: a robust preprocessing pipeline for functional MRI. *Nat Methods.* 2019;16(1):111-116.
11. Avants BB, Epstein CL, Grossman M, Gee JC. Symmetric diffeomorphic image registration with cross-correlation: evaluating automated labeling of elderly and neurodegenerative brain. *Medical image analysis.* 2008;12(1):26-41.
12. Zhang Y, Brady JM, Smith S. Hidden Markov random field model for segmentation of brain MR image. Paper presented at: Medical Imaging: Image Processing2000.
13. Greve DN, Fischl B. Accurate and robust brain image alignment using boundary-based registration. *Neuroimage.* 2009;48(1):63-72.
14. Pruim RHR, Mennes M, van Rooij D, Llera A, Buitelaar JK, Beckmann CF. ICA-AROMA: A robust ICA-based strategy for removing motion artifacts from fMRI data. *Neuroimage.* 2015;112:267-277.
15. Witten DM, Tibshirani R, Hastie T. A penalized matrix decomposition, with applications to sparse principal components and canonical correlation analysis. *Biostatistics.* 2009;10(3):515-534.

© 2024 Zhao K et al. *JAMA Network Open*.
