# Supplementary material for: Dementia Subtypes Defined Through Neuropsychiatric Symptom–Associated Brain Connectivity Patterns
Source: JAMA Netw Open. 2024 Jul 8;7(7):e2420479. doi: 10.1001/jamanetworkopen.2024.20479 (PMC11231801; doi:10.1001/jamanetworkopen.2024.20479)
Supplement: Supplement 2. — Data Sharing Statement [file jamanetwopen-e2420479-s002.pdf]

Data Sharing Statement

Zhao. Dementia Subtypes Defined Through Neuropsychiatric Symptom–Associated Brain Connectivity Patterns. *JAMA Netw Open*. Published July 8, 2024. doi:10.1001/jamanetworkopen.2024.20479

Data
Data available: No

Additional Information
Explanation for why data not available: The OASIS-3 dataset is publicly available (https://www.oasis-brains.org/). The ADNI dataset is publicly available (https://adni.loni.usc.edu/).
